# Adjunctive middle meningeal artery embolization for non-acute subdural hematoma: A GRADE-assessed meta-analysis and trial sequential analysis on randomized trials

**DOI:** 10.1007/s00701-025-06574-9

**Published:** 2025-06-02

**Authors:** Mohamed S. Elgendy, Mohamed Rifai, Amira M. Taha, Mohamed A. Faheem, Hosam I. Taha, Mostafa Meshref, Mariam Elewidi, Mohamed Abuelazm

**Affiliations:** 1 Faculty of Medicine, Tanta University, Tanta, Egypt; 2 Faculty of Medicine, Menoufia University, Shebin El Kom, Egypt; 3 Faculty of Medicine, Fayoum University, Fayoum, Egypt; 4 Neurology, Faculty of Medicine, Al-Azhar University, Cairo, Egypt

**Keywords:** Brain injury, Clinical trial, Embolization, Neurovascular, Subdural hematoma, Trauma

## Abstract

**Background and Purpose:**

Non-acute subdural hematoma (NASDH) is a prevalent neurological condition, encompassing chronic and subacute types. Despite standard-care, including surgical evacuation and medical management, recurrence rates remain high. Emerging evidence suggests that middle meningeal artery embolization (MMAE) as an adjunctive procedure may reduce recurrence. This study evaluates the efficacy and safety of MMAE in NASDH.

**Methods:**

A systematic review and meta-analysis of randomized controlled trials (RCTs) retrieved from PubMed, EMBASE, WOS, Scopus, and Cochrane until November 2024. The analysis presented risk ratios (RR) for dichotomous outcomes and mean differences (MD) for continuous outcomes, with 95% confidence intervals (CI) using R software. The GRADE system assessed evidence certainty, alongside trial sequential analysis for result reliability. PROSPERO ID: CRD42024625504.

**Results:**

Six RCTs and 1,544 patients were included, with an average of 4.7 months follow-up. Adjunctive MMAE, compared to standard-care, significantly reduced hematoma recurrence (8% vs 15.6%; RR: 0.52; 95% CI: [0.37:0.73];* P* < 0.01) and surgical rescue (4.5% vs. 12.7%; RR: 0.36; 95% CI: [0.25:0.53]; *P* < 0.01). However, no significant effect was found for recurrence without surgery (*P* = 0.94), hematoma volume (*P* = 0.18), thickness (*P* = 0.34), or hospital stay (*P* = 0.37). Infection rates were higher with MMAE (8.4% vs. 4.8%; RR: 1.81; 95% CI: [1.23:2.66]; *P* < 0.01), but adverse events (AEs), serious AEs, intracranial hemorrhage, stroke, and mortality showed no significant differences.

**Conclusion:**

Adjunctive MMAE reduced hematoma recurrence and surgical rescue rates in NASDH with an acceptable safety profile despite increased infection rates. However, further large-scale trials with extended follow-ups are needed.

**Supplementary Information:**

The online version contains supplementary material available at 10.1007/s00701-025-06574-9.

## Introduction

Subdural hematoma (SDH) is a common neurosurgical condition, particularly in the elderly, and is typically classified into acute, subacute, and chronic forms based on the timing of symptom onset. Typically, subacute SDH develops gradually between 4 and 20 days post-injury, while chronic SDH (cSDH) manifests after 20 days, both are collectively termed, based on time, as non-acute subdural hematomas (NASDH). [25, 8]

Although definitive guidelines for treating subacute and chronic SDH are lacking, surgical evacuation—typically performed through burr-hole drainage or open craniotomies—remains the standard approach for extensive and symptomatic hematomas. Conversely, supportive medical care (observation) is generally preferred for smaller collections, particularly in frail patients with multiple risk factors [11, 21]. However, Recurrence rates after surgery are reported to be approximately 13%, [19] leading to a retreatment process that causes clinical deterioration, prolonged hospital stays, and increased morbidity, mortality, and healthcare costs. [21, 19, 24]

To reduce rates of complications and recurrence associated with subacute and chronic SDH treatment, middle meningeal artery embolization (MMAE) has recently emerged with growing evidence as an alternative or adjunctive treatment to surgery or medical treatment, showing promise in reducing recurrence rates by targeting the inflammatory and vascular processes that sustain hematoma growth. [1, 17].

While recent meta-analyses and an umbrella review have reported promising results for MMAE, [26, 13, 27, 15, 31] most analyses have relied on observational data, including few randomized controlled trials (RCTs) with small sample sizes available. Our meta-analysis stands as the first to focus exclusively on RCTs, integrating the most up-to-date evidence to provide definitive comparative outcome results of MMAE versus standard-care for NASDH.

## Methodology

### Protocol registration

This systematic review and meta-analysis were conducted following the PRISMA (Preferred Reporting Items for Systematic Reviews and Meta-Analyses) guidelines (Supplementary Table 1) [23] and the Cochrane Handbook for Systematic Reviews and Meta-Analyses [12]. Our protocol has been registered in the PROSPERO database under ID: CRD42024625504.

### Data sources and search strategy

A systematic literature search was independently conducted by two authors (M.S.E. and H.I.T.) across five databases, including PubMed, Web of Science, Scopus, Cochrane, and EMBASE, up to November 23, 2024. No restrictions were applied regarding language or publication date. Our search strategy combined both free-text keywords and MeSH terms, using these terms: ("non-acute subdural hematoma") AND ("middle meningeal artery embolization") AND ("Clinical Trial"). M.S.E. also screened reference lists of relevant reviews for additional RCTs and randomly searched Google Scholar and ResearchGate to ensure comprehensive coverage. Our search strategy and literature search are detailed in Supplementary Table 2.

### Eligibility criteria

We included exclusively RCTs that met the following PICO criteria:

- **Population (P)**: adult patients (≥ 18 years) with NASDH, including both subacute and chronic cases, who required surgical evacuation.
- **Intervention (I)**: MMAE as an adjuvant treatment to standard-care, which could be surgical or supportive medical care (observation), for NASDH.
- **Comparison (C)**: standard-care without MMAE.
- **Outcomes (O)**: the primary outcomes were total recurrence, surgical rescue (composite of recurrence or progression leading to surgery), and recurrence without surgery. Secondary outcomes included: *(a) efficacy outcomes*: change in hematoma volume (ml), change in hematoma thickness (mm), hospital stay length, and functional outcomes, as assessed by modified Rankin Scale (mRS) scores of 0–2 or 0–3; and *(b) safety outcomes*: total adverse events (AEs), total serious AEs (SAEs), intracranial hemorrhage, stroke, infections and infestations, deaths from any cause within 90 days, disease-related death at 90 days, and neurologic death at 180 days.

No restrictions on sex, ethnicity, language, or publication date were applied. Additionally, we excluded animal studies, non-randomized trials, single-arm trials, feasibility studies, observational studies, reviews, books, and theses. Studies with duplicate or missing data, conference abstracts, protocols, and letters were also excluded.

### Study selection

The review screening process was conducted using Covidence, an online software tool, where duplicates were first removed. Two independent reviewers (M.A.F. and M.S.E.) then screened the remaining records based on their titles and abstracts. Studies that met the inclusion criteria were subsequently assessed through full-text screening, following the predefined PICO criteria. Any disagreements were resolved through discussion.

### Data extraction

Four reviewers (M.A.F., M.S.E., H.I.T., M.E.), at least two independently for each section, extracted the data using a pre-designed sheet on Excel (Microsoft, USA). The extracted data was organized into three key sections: (1) summary (study ID, study design, country, sample size, study arms, embolic agents, MMAE time, inclusion criteria, primary outcome, and maximum follow-up period). (2) baseline characteristics of patients: (demographic data, past medical history, hematoma details). (3) outcomes data in two sections: the efficacy (recurrence rate, surgical rescue, recurrence without surgery, change in hematoma volume, change in hematoma thickness, hospital stay length, and functional outcomes assessed by mRS scores of 0–2 or 0–3); for the safety outcomes (AEs, SAEs, intracranial hemorrhage, stroke, infections and infestations, deaths (from any cause at 90 days, disease-related at 90 days, and neurological at 180 days)).

Only the adjusted data were included in our analysis for studies that reported unadjusted and adjusted results. Ng et al. [22] calculated changes in hematoma volume and thickness using differences from post-surgical measurements, unlike other studies that used baseline measurements after randomization.

### Risk of bias and certainty of evidence

The quality assessment of the included RCTs was evaluated using the Cochrane Risk of Bias (RoB) 2 tool [28], which assesses five key areas: randomization biases, deviations from the intended intervention, missing outcome data, inconsistencies in outcome measurement, and selective reporting. Then the studies were classified as having a “low risk”, “some concerns”, or “high risk” of bias. Based on these criteria, studies were classified as having a"low risk,""some concerns,"or"high risk"of bias. Two reviewers (M.E. and M.A.F.) performed this assessment, resolving disagreements by a third reviewer (M.S.E.). Although publication bias was considered, it could not be formally evaluated due to the inclusion of fewer than ten studies [28].

The certainty of the evidence was assessed by M.S.E. using the GRADE system (Grading of Recommendations, Assessment, Development, and Evaluation) [10]. The quality of the evidence was categorized as “high”, “moderate”, “low”, or “very low” certainty.

### Statistical analysis

The study employed R version 4.3, using the Meta, Metafor, and Dmetar packages for statistical analysis. The analysis combined results from multiple studies using either risk ratios (for dichotomous outcomes) or mean differences (for continuous outcomes), both with 95% confidence intervals. Additionally, Continuity correction was applied for all zero events following the Cochrane Handbook's guidelines for handling rare events. [12] A random-effects model was applied when significant heterogeneity (I^2^ > 50%) was detected using the Chi-square and I-square tests; otherwise, a common-effect model was used.

Heterogeneity was interpreted according to the Cochrane Handbook (chapter nine) [12], with an I^2^ value of 0–40 percent indicating low heterogeneity, 30–60 percent signifying moderate heterogeneity, 50–90 percent may represent substantial heterogeneity, and 75–100 percent signifying considerable heterogeneity. A Chi-square test p-value below 0.1 was considered statistically significant for heterogeneity.

#### Sensitivity analysis and subgrouping

To address heterogeneity and ensure the robustness of the results, sensitivity analyses were conducted by excluding individual studies to assess their influence on the overall findings. A leave-one-out analysis was performed, sequentially excluding each study from the meta-analysis to identify any influential studies disproportionately affecting the pooled effect estimates. Additionally, a subgroup analysis based on the difference in the intervention protocol was conducted on applicable outcomes to explore the impact of different techniques on the outcomes. The studies were stratified into two subgroups: MMAE with surgery only and MMAE with surgery or medical treatment.

#### Trial sequential analysis

We used Trial Sequential Analysis (TSA) to ensure our meta-analysis had enough data to evaluate intervention effectiveness. Further studies are unnecessary if the analysis curve crosses the conventional boundary and the trial sequential monitoring boundary (TSMB). Otherwise, more research is needed. Our parameters included a 0.05 significance level, 80% power, and an expected 20% risk reduction for dichotomous outcomes [30].

#### Number needed to treat

The number needed to treat (NNT) was calculated as the reciprocal of the absolute risk reduction (ARR) derived from surgical rescue rates, with confidence intervals computed using the Wilson score method by Clincalc online calculator.

## Results

### Study selection

**Fig. 1:**
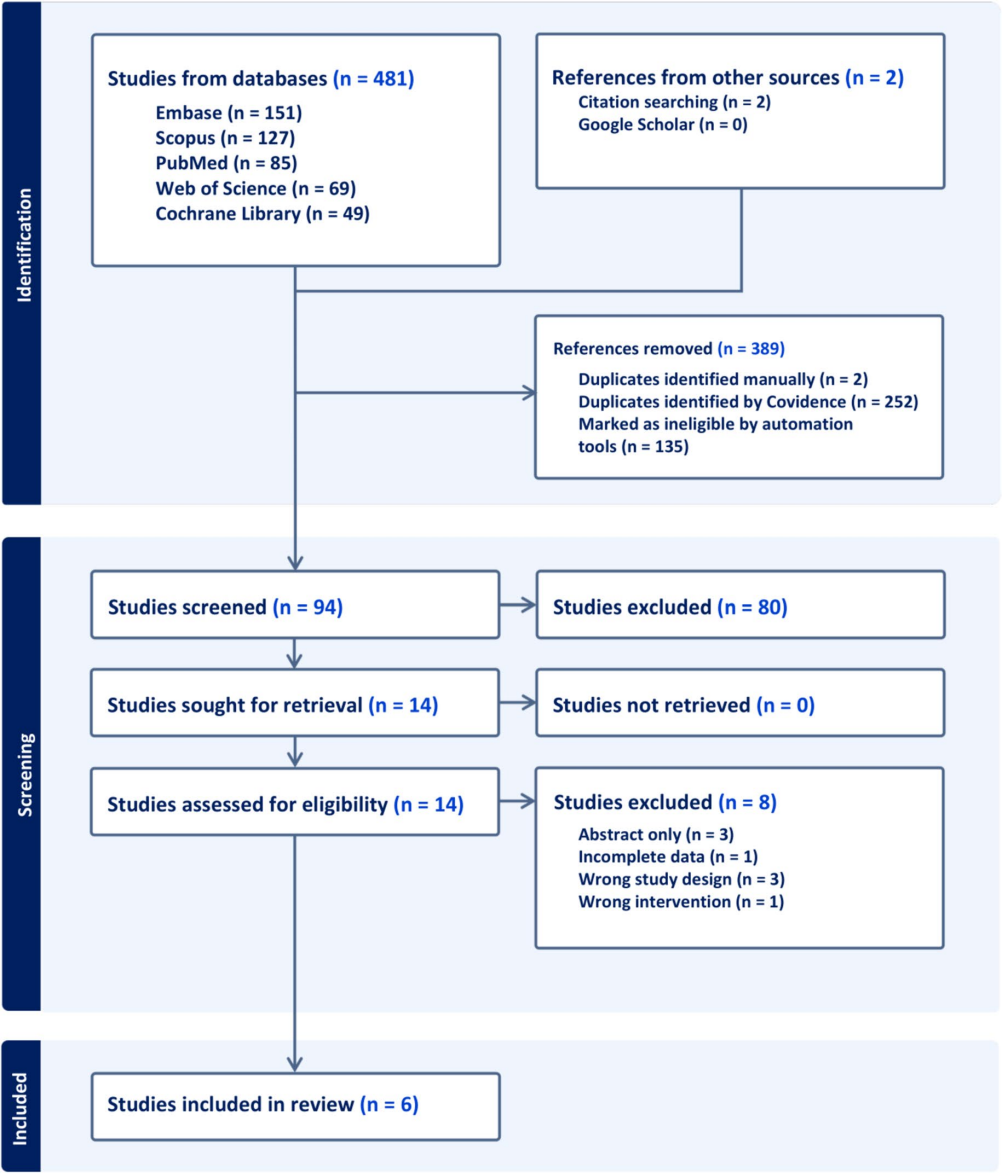
PRISMA flow chart for the systematic search and selection process

Our search identified 483 records. After removing 135 duplicates and exclusions via Covidence software, 94 unique studies remained. Eighty studies were excluded during the title and abstract screening. The remaining fourteen studies underwent full-text review with eight excluded (Supplementary Table 3) and leaving six RCTs [22, 16, 4, 9, 3, 18] as the final inclusion. The PRISMA flow diagram summarizes the selection process in Fig. 1.

### Characteristics of included studies

The final selection included six RCTs comprising 1,544 patients, 758 in the MMAE group and 785 in the standard-care group, with an average follow-up of 4.7 months. Notably, Liu et al. (MAGIC-MT) [18] and Davies et al. (EMBOLISE) [3] included both subacute and chronic SDH in their trials, whereas the remaining studies exclusively included cSDH​​. The mean age across the included studies was 70.46 years (± 8.5). The study populations were predominantly male (76%). A detailed summary of the included trials and patient characteristics is provided in Table 1.

**Table 1:** Summary overview of the included trials

Study ID	Study design	Country (No of centers)	Sample size, no	Study arms		Embolic Agents	MMAE timing	Inclusion Criteria				Primary outcome	MFUP, months
				**Intervention**	**Control**			**Age, Y**	**mRS**	**SDH thickness**	**Condition**		
**Davies et al. 2024 (EMBOLISE)** [3]	Multicenter, open-label, RCT	USA (39)	400	MMAE + Surgery	Surgery alone	Onyx	Both before & after surgery	18:90	0:3	More than 15 mm	Symptomatic subacute or chronic SDH requiring surgery	Recurrence or progression of SDH led to repeat surgery at 90 days	6
**Liu et al. 2024 (MAGIC-MT)** [18]	Multicenter, open-label, RCT	China (31)	722	MMAE + (Medical TTT or Surgery)	Medical TTT ± surgery	Onyx	Before surgery	≥ 18	0:2	More than 10 mm	Symptomatic subacute or chronic SDH requiring surgery	Recurrence or progression of SDH at 90 days	3
**Fiorella et al. 2024 (STEM)** [9]	Multicenter, open-label, RCT	USA, France, Spain, Germany (32)	310	MMAE + (Medical TTT or Surgery)	Medical TTT or surgery alone	Squid	Before surgery	Adults	NM	More than 10 mm	Symptomatic chronic SDH requiring surgery or Medical TTT	Recurrent or residual SDH at 180 days	6
**Debs et al. 2024 (Endovascular)** [4]	Monocenter, open-label, RCT	USA	35	MMAE + Surgery	Surgery alone	Onyx	After surgery	18:90	0:4	More than 10 mm	Symptomatic chronic SDH requiring surgery	Recurrence of SDH requiring reoperation	7
**Lam et al. 2023** [16]	Monocenter, open-label, RCT	Australia	36	MMAE + Surgery	Surgery alone	Squid-12, Onyx-18, & others^#^	After surgery	≥ 18	0:6	More than 10 mm	Symptomatic chronic SDH requiring surgery	Symptomatic recurrence requiring reoperation	3
**Ng et al. 2019** [22]	Monocenter, single-blinded, RCT	France	41	MMAE + Surgery	Surgery alone	Polyvinyl alcohol	After surgery	≥ 18	NM	More than 10 mm	Symptomatic de novo chronic SDH requiring surgery	Hematoma volume resorption at 90 days CT scan	3

(# Others: refers to Phil 25% and 25% n-butyl cyanoacrylate with lipoidol), No Number, MMAE middle meningeal artery embolization, Y year, mRS modified Rankin scale, MFUP maximum follow up period, RCT randomized controlled trial, TTT treatment; NM not mentioned, SDH subdural hematoma

The mean age of participants across the included studies was 70.46 years (± 8.5). The study populations were predominantly male, with 76% male participants and 24% female. Among the participants, 27% were on antiplatelet or anticoagulant therapy. Additionally, 60.3% of participants had a history of hypertension in the relevant studies [9, 3, 18]. Detailed characteristics of included patients are provided in Table 2 and Supplementary Table S4.

**Table 2:** Baseline characteristics of the included patients

Study ID	Groups	No. of patients	Age, y. M. (SD)	Gender; male, no. (%)	Smoking, no. (%)	Alcoholism, no. (%)	HTN, no, (%)	Antiplatelet or anticoagulant, no. (%)	Head trauma, no. (%)
**Davies et al. 2024 (EMBOLISE)** [3]	**Adjunctive MMAE**	197 (49.3)	73.0 ± 11	143 (72.6)	19 (9.6)	20 (10.2)	150 (76.1)	75 (38.1)	NM
	**Standard-care**	203 (50.7)	71.0 ± 11.3	149 (73.4)	26 (12.8)	27 (13.3)	151 (74.4)	79 (38.9)	NM
**Liu et al. 2024 (MAGIC-MT)** [18]	**Adjunctive MMAE**	360 (49.9)	67.7 ± 2.4	292 (81.1)	57 (15.8)	46 (12.8)	162 (45.0)	51 (14.1)	178 (49.4)
	**Standard-care**	362 (50.1)	69 ± 2.4	304 (84.0)	57 (15.7)	48 (13.3)	166 (45.9)	53 (14.6)	201 (55.5)
**Fiorella et al. 2024 (STEM)** [9]	**Adjunctive MMAE**	149 (48)	72.8 ± 10.4	97 (65)	15 (10)	15 (10)	110 (74)	56 (38)	NM
	**Standard-care**	161 (52)	73.4 ± 11.3	119 (74)	14 (9)	15 (9)	125 (78)	67 (42)	NM
**Debs et al. 2024 (Endovascular)** [4]	**Adjunctive MMAE**	17 (48.5)	66.1 ± 12	12 (70.6)	NM	NM	NM	6 (35)	NM
	**Standard-care**	18 (51.4)	70.8 ± 9.4	11 (61.1)	NM	NM	NM	7 (39)	NM
**Lam et al. 2023** [16]	**Adjunctive MMAE**	17 (45.7)	64.2	12 (75)	NM	NM	NM	3 (18.75)	13 (81.3)
	**Standard-care**	19 (54.3)	72.4	11 (57.8)	NM	NM	NM	4 (21.1)	16 (84.2)
**Ng et al. 2019** [22]	**Adjunctive MMAE**	19 (46)	77.4 ± 11	10 (52.6)	NM	3 (16)	NM	7 (37)	12 (63)
	**Standard-care**	22 (54)	74.7 ± 14	13 (59.1)	NM	3 (14)	NM	9 (41)	15 (68)

Data are presented in mean ± SD or proportions as (%). *No* Number; *M* mean; *SD* standard deviation, *MMAE* middle meningeal artery embolization, *HTN* hypertension; *NM* not mentioned

### Risk of bias and certainty of evidence

Out of the six included trials assessed using the RoB 2 tool, four [16, 9, 3, 18] demonstrated an overall low risk across all domains. Despite the open-label design, the reliance on objective clinical and radiological outcomes effectively minimized subjective influence. The trials generally exhibited robust randomization processes, high adherence to intervention protocols, and minimal missing outcome data. However, two studies deviated from this trend. Debs et al. [4] and Ng et al. [22] were flagged with some concerns or high risk due to issues in randomization (D1) and missing patients (D3) post-randomization without appropriate analysis. The RoB diagram summarizes the assessment in Supplementary Fig. S1.

All GRADE-assessed outcomes were rated as having moderate to low certainty of evidence. All GRADE assessment details are provided in Table 3 and Supplementary Table S5.

**Table 3:** Grading of recommendations assessment, development, and evaluation (GRADE) evidence profile

Certainty assessment							Study event rates (%)		Effect		Certainty
**№ of studies**	**Study design**	**RoB**	**Inconsistency**	**Indirectness**	**Imprecision**	**Others**	**MMA embolization**	**Standard-care**	**Relative** **(95% CI)**	**Absolute (95% CI)**	
**Total Recurrence Rate**											
4	RCTs	not serious	not serious	not serious	serious^a^	none	41/515 (8.0%)	83/532 (15.6%)	**RR 0.52** (0.37 to 0.73)	**75 fewer per 1,000** (from 98 to 42 fewer)	⨁⨁⨁◯ Moderate^a^
**Surgical Rescue (Recurrence or Progression Led to Surgery)**											
6	RCTs	not serious	not serious	not serious	serious^a^	none	33/729 (4.5%)	96/753 (12.7%)	**RR 0.36** (0.25 to 0.53)	**81 fewer per 1,000** (from 96 to 60 fewer)	⨁⨁⨁◯ Moderate^a^
**Recurrence without Surgery**											
3	RCTs	not serious	not serious	not serious	very serious^a,b^	none	17/496 (3.4%)	17/510 (3.3%)	**RR 1.03** (0.53 to 1.97)	**1 more per 1,000** (from 16 fewer to 32 more)	⨁⨁◯◯ Low^a,b^
**Change in Hematoma Thickness (mm)**											
4	RCTs	not serious	not serious	not serious	serious^c^	none	565	585	-	MD **0.06 mm lower** (0.17 lower to 0.06 higher)	⨁⨁⨁◯ Moderate^c^
**Infections and Infestations**											
4	RCTs	not serious	not serious	not serious	Serious^a,d^	none	60/717 (8.4%)	36/750 (4.8%)	**RR 1.81** (1.23 to 2.66)	**39 more per 1,000** (from 11 to 80 more)	⨁⨁◯◯ Low^a,d^
**Total Adverse Events (AEs)**											
4	RCTs	not serious	not serious	not serious	serious^e^	none	265/717 (37.0%)	271/750 (36.1%)	**RR 1.05** (0.93 to 1.18)	**18 more per 1,000** (from 25 fewer to 65 more)	⨁⨁⨁◯ Moderate^e^
**Total Serious AEs (SAEs)**											
3	RCTs	not serious	serious^f^	not serious	serious^e^	none	151/701 (21.5%)	184/731 (25.2%)	**RR 0.88** (0.68 to 1.13)	**30 fewer per 1,000** (from 81 fewer to 33 more)	⨁⨁◯◯ Low^e,f^
**Deaths from Any Cause Within 90 Days**											
5	RCTs	not serious	not serious	not serious	serious^a,e^	none	19/736 (2.6%)	20/771 (2.6%)	**RR 1.02** (0.56 to 1.86)	**1 more per 1,000** (from 11 fewer to 22 more)	⨁⨁◯◯ Low^a,e^

*CI* confidence interval, *MD* mean difference, *RR* risk ratio, *RoB* risk of bias, *MMA* middle meningeal artery, *RCT* randomized controlled trial

**Explanations**

a. Low number of events (less than 200)

b. Wide CI with crossing no effect line (1.0), not exclude the risk of appreciable benefit/harm

c. Cross the line of no effect (0), not exclude the risk of appreciable benefit/harm

d. Wide CI, which may not exclude the risk of appreciable benefit/harm

e. Cross no effect line (1.0), not exclude the risk of appreciable benefit/harm

f. I^2 ≥ 50%; shows significant heterogeneity

### Primary outcomes: recurrence & surgical rescue

**Fig. 2:**
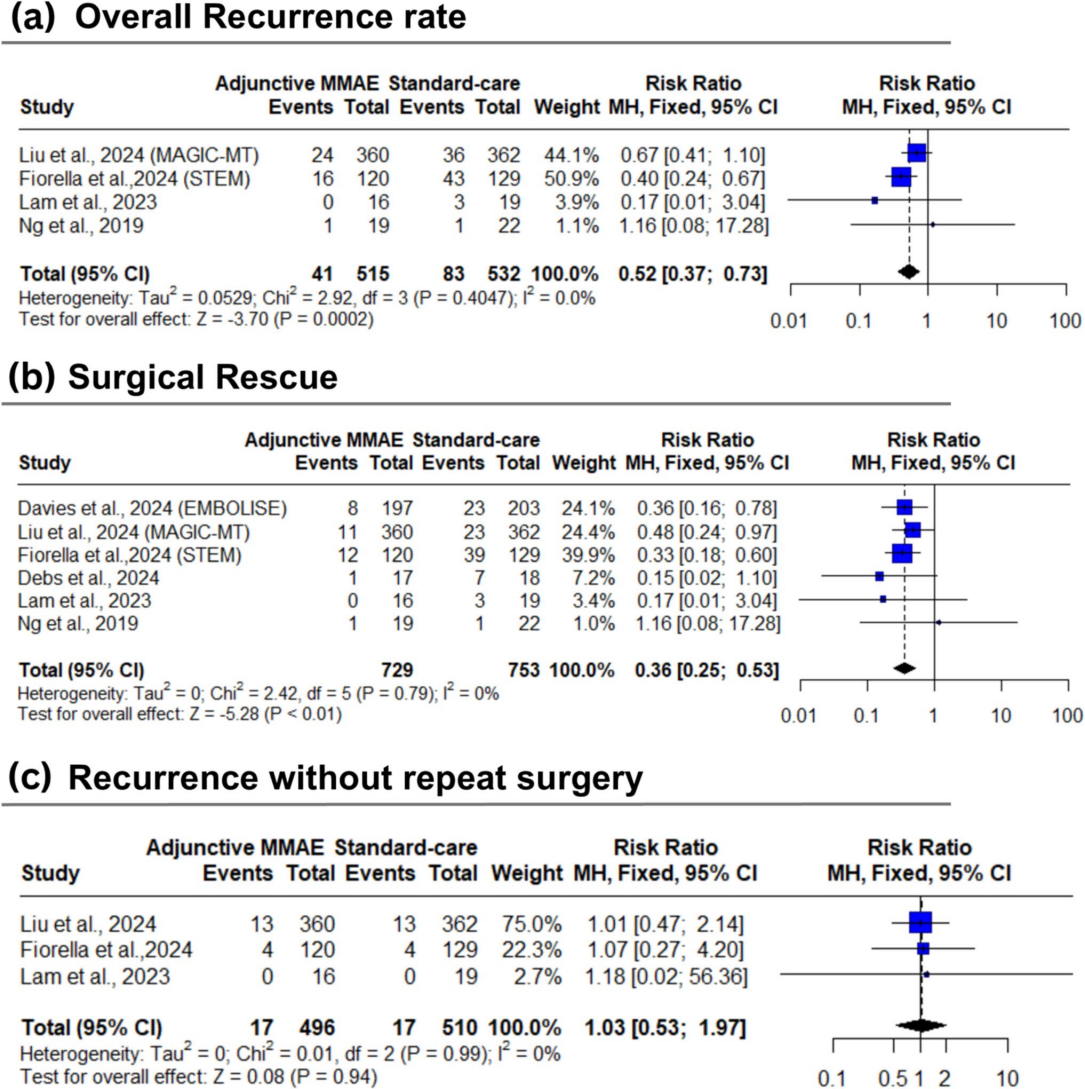
Forest plot for clinical efficacy outcomes. *MMAE* middle meningeal artery embolization, *MH* Mantel–Haenszel, *CI* confidence interval

Compared to the standard care group, the adjunctive MMAE group was significantly associated with low rates for total recurrence of hematoma (8.8% vs 18.3%; RR: 0.52; 95% CI [0.37 to 0.73]; *P* < 0.01) [moderate certainty] (Fig. 2a). Also, the MMAE group had a significant reduction than the standard care group in surgical rescue (4.5% vs 12.7%; RR: 0.36; 95% CI: [0.25 to 0.53]; *P* < 0.01) [moderate certainty] (Fig. 2b). The test for subgroup differences based on MMAE either with surgery alone or with surgery and medical treatment was insignificant for both total recurrence (*P* = 0.7498) (Supplementary Fig. S2a) and surgical rescue (*P* = 0.6433) (Supplementary Fig. S2b).

**Fig. 3:**
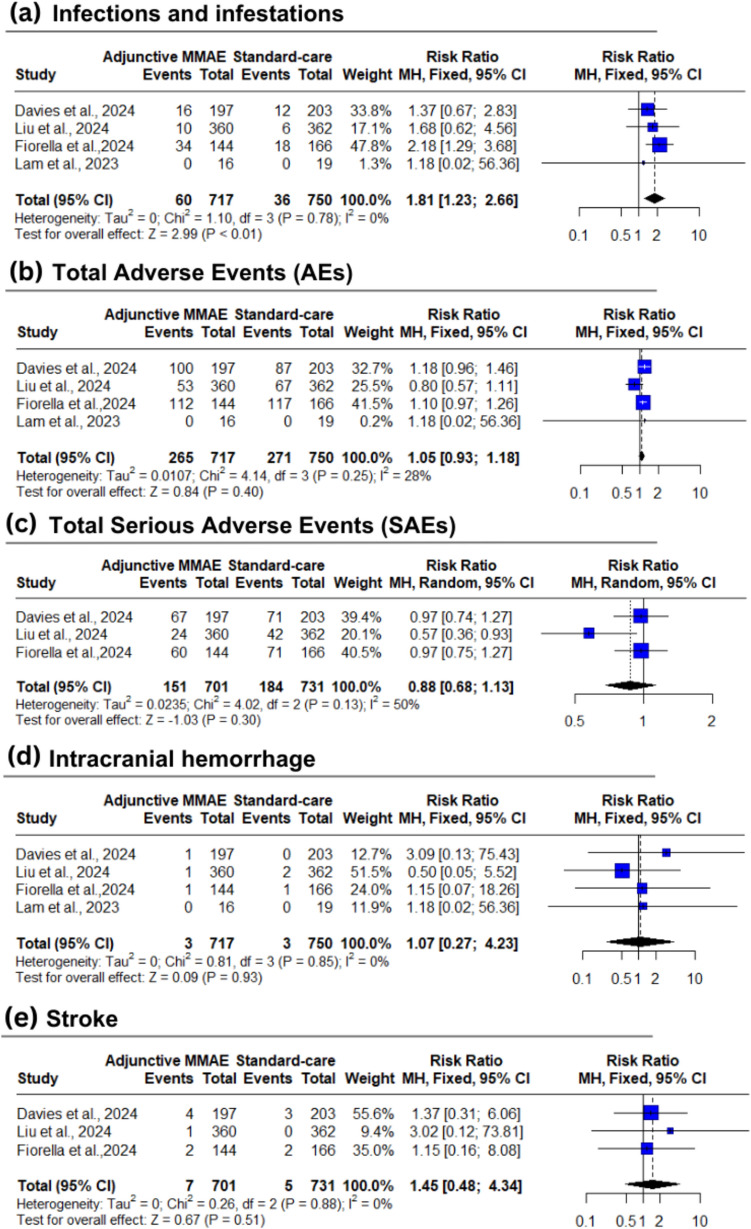
Forest plot for rates of infections and infestations, total adverse events (AEs), total serious AEs, intracranial hemorrhage, and stroke. *MMAE* middle meningeal artery embolization, *MH* Mantel–Haenszel, *CI* confidence interval

However, no significant difference was observed in the incidence of recurrence without repeat surgery between adjunctive MMAE and standard care groups (3.4% vs 3.3%; RR: 1.03; 95% CI: [0.53 to 1.97]; *P* = 0.94) [low certainty] (Fig. 3c).

The pooled studies were homogenous for the total recurrence rate of hematoma (I^2^ = 13.3%, *P* = 0.326), surgical rescue (I^2^ = 0%, *P* = 0.79), and recurrence without repeat surgery (I^2^ = 0%, *P* = 0.99) (Fig. 3).

Based on pooled surgical rescue rate, the ARR was 8.2% and NNT to prevent one surgical rescue was 12 (95% CI: [9 to 18]).

### Secondary outcomes

#### Efficacy outcomes

There was no significant difference between both groups in mean change of hematoma thickness (MD: −0.06 mm; 95% CI: [−0.17 to 0.06]; *P* = 0.34) [moderate certainty] (Supplementary Fig. S3a), mean change of hematoma volume (MD: −0.16 ml; 95% CI: [−0.40 to 0.07]; 0.18) [low certainty] (Supplementary Fig. S3b), mean days of hospital stay (MD: 0.12 days; 95% CI: [−0.02 to 0.26]; *P* = 0.10) [moderate certainty] (Supplementary Fig. S3c), good functional Outcome (mRS 0–2) (88.6% vs 88.5%; RR: 1.00; 95% CI: [0.96 to 1.04]; *P* = 0.98) [moderate certainty] (Supplementary Fig. S4a), and favorable functional Outcome (mRS 0–3) (95.5% vs 94.7%; RR: 1.01; 95% CI [0.98 to 1.03]; *P* = 0.58) [moderate certainty] (Supplementary Fig. S4b).

The pooled studies were homogenous in mean change of hematoma thickness (I^2^ = 0%, *P* = 0.95), good functional Outcome (mRS 0–2) (I^2^ = 0%, *P* = 0.73), and mean days of hospital stay (I^2^ = 0%, *P* = 0.37). However, pooled studies showed moderate heterogeneity in favorable functional outcome (mRS 0–3) (I^2^ = 46%, *P* = 0.16). Considerable heterogeneity in mean change of hematoma volume (I^2^ = 62%, *P* = 0.07) (Supplementary Fig. S3, S4). For the mean change of hematoma volume, heterogeneity was best resolved after excluding Ng et al. (I^2^ = 6%) (Supplementary Fig. S5).

#### Safety outcomes

The adjunctive MMAE group showed a higher rate of infections compared to the standard care group (8.4% vs 4.8%; RR: 1.81; 95% CI: [1.23 to 2.66]; *P* < 0.01) [low certainty] (Fig. 3a). On subgrouping, there was no significant difference (*P* = 0.3519) (Supplementary Fig. S6a).

**Fig. 4:**
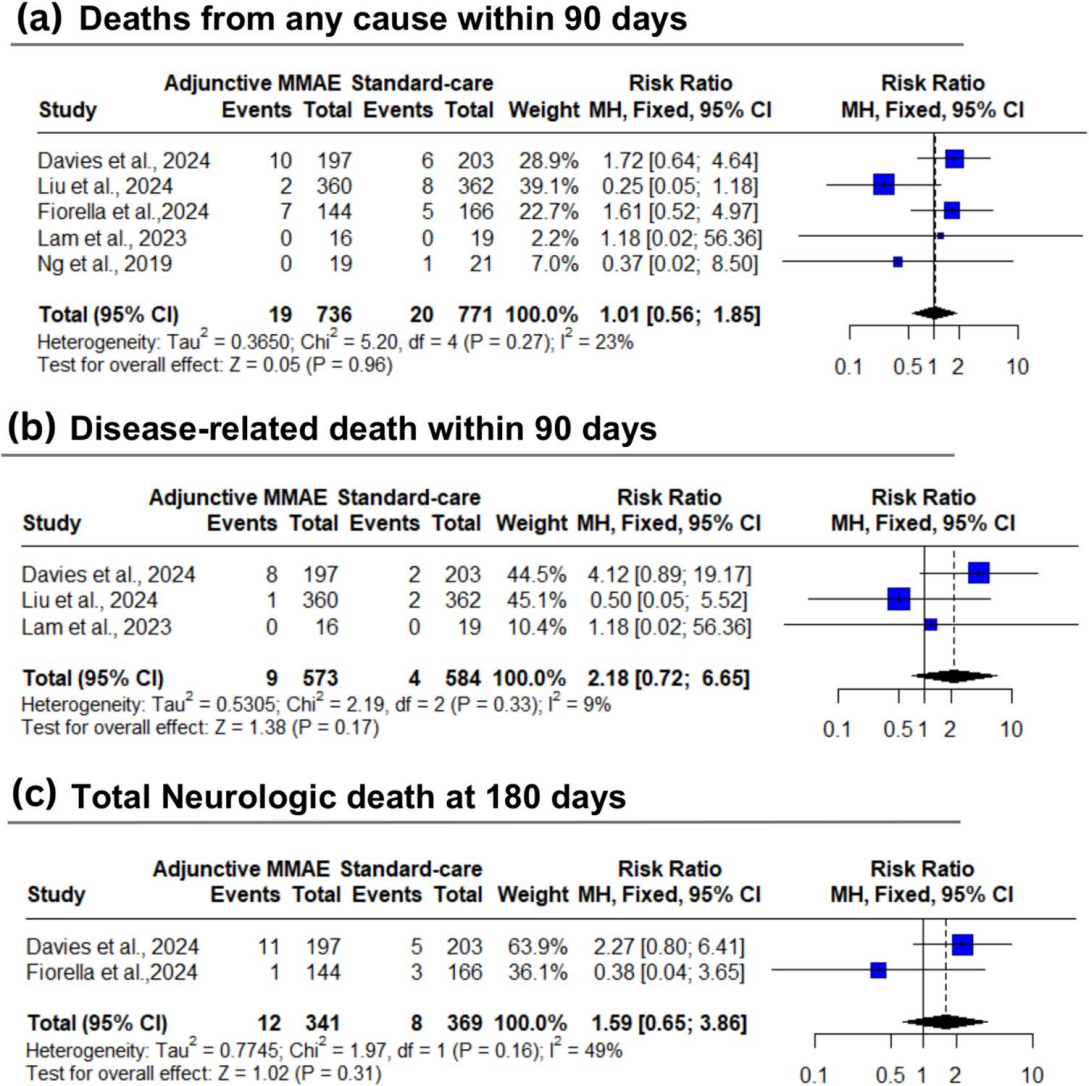
Forest plot for rates of death (any cause, disease-related, and neurological causes). *MMAE* middle meningeal artery embolization, *MH* Mantel–Haenszel, *CI* confidence interval

However, compared to the standard care group, adjunctive MMAE group showed no significant differences in total AEs (37% vs 36.1%; RR: 1.06; 95% CI: [0.90 to 1.26]; *P* = 0.41) [moderate certainty] (Fig. 3b), total SAEs (21.5% vs 25.2%; RR: 0.88; 95% CI: [0.68 to 1.13]; *P* = 0.30) [low certainty] (Fig. 3c), intracranial hemorrhage (0.4% vs 0.4%; RR: 1.07; 95% CI: [0.27 to 4.23]; *P* = 0.93) [low certainty] (Fig. 3d), stroke (1.0% vs 0.7%; RR: 1.45; 95% CI: [0.48 to 4.34]; *P* = 0.51) [low certainty] (Fig. 3e), deaths from any cause within 90 days (2.6% vs 2.6%; RR: 1.01; 95% CI: [0.56 to 1.85]; *P* = 0.96) [moderate certainty] (Fig. 4a), disease-related death at 90 days (1.6% vs 0.7%; RR: 1.80; 95% CI: [0.38 to 8.50]; *P* = 0.17) [low certainty] (Fig. 4b), and total Neurologic death at 180 days (3.5% vs 2.2%; RR: 1.59; 95% CI: [0.65 to 3.86]; *P* = 0.31) [low certainty] (Fig. 4c).

The pooled studies were homogenous in intracranial hemorrhage (I^2^ = 0%, *P* = 0.85), stroke (I^2^ = 0%, *P* = 0.88), and infections and infestations (I^2^ = 0%, *P* = 0.78) with low heterogeneity in disease-related death at 90 days (I^2^ = 9%, *P* = 0.33), deaths from any cause within 90 days (I^2^ = 23%, *P* = 0.27), and total AEs (I^2^ = 28%, *P* = 0.13). However, pooled studies showed moderate heterogeneity in total SAEs (I^2^ = 50%, *P* = 0.13) and total neurologic death at 180 days (I^2^ = 49%, *P* = 0.16) (Fig. 3, 4). Heterogeneity was best resolved for total SAEs after excluding Liu et al. (I^2^ = 0%) (Supplementary Fig. S7).

The test for subgroup analysis was insignificant in total AEs (*P* = 0.1568) (Supplementary Fig. S6b), intracranial hemorrhage (*P* = 0.4640) (Supplementary Fig. S8a), and total death within 90 days (*P* = 0.2984) (Supplementary Fig. S8b).

### Trial Sequential Analysis

TSA showed a significant difference in favor of adjunctive MMAE over standard care, as the cumulative z-score crossed the TSMB and the conventional monitoring boundary for primary outcomes: total recurrence (Supplementary Fig. S9) and surgical rescue (Supplementary Fig. S10). This indicates that current evidence is sufficient and conclusive, and no further trials are needed.

## Discussion

Our meta-analysis synthesized evidence from six recent RCTs involving 1,544 patients to evaluate the efficacy and safety of MMAE as an adjuvant treatment for non-acute SDH (chronic and subacute types). The results consistently demonstrated that adjunctive MMAE significantly reduced recurrence rates, decreasing the need for further surgical intervention. While there was no significant difference in recurrence rates without re-surgery between the MMAE and standard care groups, overall recurrence rates differed significantly, with 8% and 15.6%, respectively. Surgical rescue rates were also markedly lower with MMAE, at 4.5%, versus 12.7% with standard care.

Similarly, safety results between MMAE and standard treatment were comparable, except for a significantly increased infection rate in the MMAE group. The current study highlights MMAE's potential as a successful NASDH adjunctive intervention, with moderate evidence to support its ability to lower the number of recurrence-related surgical procedures without considerably compromising safety.

A more widely accepted concept of the pathophysiology of cSDH development involves chronic inflammation and neovascularization, replacing the old theory of cortical bridging vein rupture and gradual venous hemorrhage. According to this recent theory, following an initial injury to dural border cells, an inflammatory cycle including macrophage infiltration, granulation tissue formation, and membrane development results in angiogenesis and the construction of connections with perforators that are triggered by MMAE. [5, 6] Chronic microhemorrhages that prolong the cycle are caused by blood leaking from fragile vessels created during this phase. [6, 20] The increase in cSDH is caused by an imbalance between blood accumulation and reabsorption [5, 6]. cSDH is associated with significant morbidity, mortality, and additional healthcare burden [21]. Current surgical or medical treatments provide moderate benefits with reported side effects. [21, 24]

Due to a lack of alternative therapy, recurrence rates of up to 20% and reoperation rates of up to 10% in well-managed cases have been deemed acceptable[21]. Given the proposed involvement of the MMAE in the updated pathophysiologic mechanism behind the development and maintenance of cSDH, MMAE has become more popular as a potentially effective treatment. This has been reinforced by angiographic data that indicates the post-procedure resolution of aberrant vascular patterns. [1, 17]

In our meta-analysis, surgical rescue occurred in 33/729 (4.5%) in the MMAE adjunctive group compared to 96/753 (12.7%) in the standard-care group. Similar to our findings, Zhang et al. meta-analysis found that the MMAE adjunctive group experienced considerably lower re-operation rates than surgery alone (4.4% vs. 10.9%) with no significant heterogeneity [31]. Sattari et al. reported that patients who underwent MMAE (1.1%) had lower surgical rescue than those receiving conservative management (16.1%) [26]. Furthermore, Shakir et al.'s meta-analysis of 23 observational studies concluded that MMAE, either alone or as an adjunct, significantly reduces the risk of hematoma recurrence. [27] Comparable differences between adjunctive MMAE and standard-care have been documented; however, the small sample size and low statistical power may limit the applicability of this evidence. [22, 16, 4, 9, 3, 18]

Furthermore, hospital stay duration did not significantly differ across the included trials, which may be related to the form of MMAE used as an additional intervention. [4, 18] Also, definitive treatment of cSDH is supposed to reduce hospital stay and admissions. This result is in alignment with the findings of Zhang et al. [31] and Sattari et al. [26]; however, Shakir et al. [27] found that hospital stay in the MMAE were significantly longer, regardless of whether they were used as an adjunct therapy or alone. Authors attributed this difference to the condition of selected patients for extra intervention compared to the other group.

No mortality benefit had been observed in most included trials, supporting our finding. [22, 16, 4, 9, 3, 18] Also, comparable AEs and functional outcomes further support the safety of MMAE being an additional procedure for cSDH.

## Cost-effectiveness

Between 2016 and 2020, the number of MMAEs performed in the USA increased tenfold, indicating the procedure's exponential rise. As interest surrounding recent trial results grows, there is a possibility that indications will be expanded beyond what the data warrants, which could result in the use of MMAE being unnecessary or unjustified. Cost-effectiveness may be increased by limiting MMAE to situations with significant residual collections following evacuation or early asymptomatic regrowth. [7] As reported by Liu et al., mean hospitalization costs in renminbi (RMB) were 44,440 for MMAE and 21,592 for usual-care. [18]

According to certain recent studies, [29, 2] MMAE might be less expensive than traditional surgical treatment. However, this can vary according to the protocol, especially embolic agent selection and treatment algorithm time. For instance, particles can cost anywhere from 5 to 10% as much as liquid embolic. Procedure costs could be reduced by avoiding general anesthesia and unnecessary hospital stays. Hung et al. found that 82% of patients required less than two days in the ICU, suggesting ICU may often be unnecessary post-surgery. Switching from ICU to intermediate care units (IMC) could save $1,066.53 per patient per night, while transitioning to non-monitored facilities could save $1367.94 per night. Carefully optimizing postprocedural care levels could significantly lower overall costs for MMAE in cSDH. [14]

The NNT of MMAE was approximately 13 to prevent one hematoma recurrence and 12 to avoid one surgical rescue, highlighting that its value must be weighed against procedural risk, cost, patient preference, and the severity of the clinical condition.

While an NNT of 12–13 showing some advantage, this metric must be interpreted with caution. MMAE is an invasive, resource-intensive procedure with higher upfront costs and, in our analysis, a higher rate of infections compared to surgery. Performing 12 embolizations to avoid one reoperation may not be justified, particularly when the surgery is straightforward and well-tolerated. Moreover, cost-effectiveness studies offer mixed conclusions [2, 14], and patient preferences between multiple less invasive procedures versus a single surgical intervention remain largely unexamined. The value of MMAE will depend on clinical context—patient comorbidities, hematoma characteristics, procedural risks, and institutional resources. Thus, MMAE should not be adopted universally but rather applied selectively, tailored to those most likely to benefit based on individual risk profiles.

## Strengths

To the best of our knowledge, this is the first meta-analysis exclusively focused on RCTs evaluating the safety and efficacy of MMAE for nonacute SDH. By limiting inclusion to RCTs, our study ensures a high level of evidence and minimizes biases associated with observational designs. This meta-analysis strictly adhered to PRISMA guidelines and was pre-registered in PROSPERO, ensuring transparency and methodological rigor throughout the process. Also, our analysis incorporated robust tools by the GRADE framework for evaluating the certainty of evidence across outcomes. Including six geographically diverse RCTs involving 1,544 patients from multiple countries (China, USA, France, Spain, Germany, and Australia) enhances the generalizability of our findings. Additionally, the analysis included sensitivity analyses to explore and address sources of heterogeneity, providing deeper insights into treatment effects.

## Limitations

The main limitations of this meta-analysis are as follows: (i) The relatively small number of included RCTs (*n =* 6) may have limited the strength and applicability of specific subgroup analyses. (ii) Significant heterogeneity in the intervention protocols was observed across the included studies, particularly regarding the timing of MMAE administration. While two trials [9, 18] performed MMAE before surgery, Debs et al. [4], Lam et al. [16], and Ng et al. [22] administered it after surgery, whereas EMBOLISE [3] included both pre- and post-surgery. (iii) Variability in the choice of embolic agents (Onyx, Squid, polyvinyl alcohol). (iv) Also, the control protocols, with the intervention, varied across studies. MAGIC-MT [18] and STEM [9] compared MMAE combined with medical treatment or surgery to medical treatment and/or surgery alone, whereas other studies focused exclusively on MMAE with surgery versus surgery alone. (v) The surgical procedures varied across the included studies, including burr-hole drainage, craniotomy, and subdural evacuating port system. (vi) There was also variation in patient inclusion criteria and disease severity across the studies. In contrast, EMBOLISE [3] and MAGIC-MT [18] included both subacute and chronic SDH patients; the remaining studies exclusively focused on cSDH. (vii) The follow-up periods were short limiting the assessment of long-term recurrence rates and safety outcomes. (viii) Finally, reporting AEs and SAEs items was inconsistent across the included studies, limiting the robustness of pooled safety analyses.

## Implications for future research

Future research should prioritize standardizing intervention and control protocols in MMAE for NASDH. Key areas include the timing of MMAE, the selection of embolic agents, consistent comparator definitions, surgical techniques, and clearly defined patient inclusion criteria. Additionally, studies should incorporate extended follow-up periods and consistent reporting of AEs to strengthen the evidence base for MMAE. Evaluating the long-term cost-effectiveness of MMAE is also crucial to guide clinical decision-making and inform healthcare policy.

## Conclusion

In NASDH management, adjunctive MMAE significantly reduces recurrence rates and the need for surgical rescue compared to standard care, with a generally acceptable safety profile despite a modest increase in infection risk. However, clinical decisions should carefully weigh procedural risks, costs, and individual patient factors. Key uncertainties remain—particularly regarding patient selection, timing, embolic technique, the need for concurrent surgical evacuation, and use in acute settings. Further large-scale, long-term RCTs are needed to define MMAE’s optimal role in routine practice.

## Supplementary Information

Below is the link to the electronic supplementary material.

Supplementary file1 (PDF 1309 KB)

## Data Availability

All data analyzed during this study are included in this published article and its supplementary information files. No external datasets were generated or analyzed.
