# Supplementary material for: Adjunctive middle meningeal artery embolization for non-acute subdural hematoma: A GRADE-assessed meta-analysis and trial sequential analysis on randomized trials
Source: Acta Neurochir (Wien). 2025 Jun 2;167(1):160. doi: 10.1007/s00701-025-06574-9 (PMC12129861; doi:10.1007/s00701-025-06574-9)
Supplement: Supplementary file 1 — Supplementary file1 (PDF 1309 KB) [file 701_2025_6574_MOESM1_ESM.pdf]

Supplemental Material

Title.

Adjunctive Middle Meningeal Artery Embolization for Non-Acute Subdural Hematoma: A GRADE-Assessed Meta-Analysis and Trial Sequential Analysis on Randomized Trials

The authors have provided this supplementary material to demonstrate additional information about this study.

INDEX.

Tables:

1. **Supplementary Table 1** PRISMA 2020 checklist……………..…………………..*(page 3)*
2. **Supplementary Table 2** Search strategy and literature search……………*(page 6)*
3. **Supplementary Table 3** Excluded studies with reasons in the full-text screening……………………………………………………………………..*(page 7)*
4. **Supplementary Table 4** Baseline characteristics of the included patients' hematoma lesions……………………………………………………………*(page 8)*
5. **Supplementary Table 5** Remaining for grading of recommendations assessment, development, and evaluation (GRADE) evidence profile………..……...……..*(page 9)*

Figures:

1. **Supplementary Fig. 1** Overview of the risk of bias of the included randomized controlled trials……………………………………………………………*(page 13)*
2. **Supplementary Fig. 2** Forest plot of subgrouping of overall recurrence rate and surgical rescue………………………….....……………………..…...……*(page 14)*
3. **Supplementary Fig. 3** Forest plot of radiological efficacy outcomes and hospital stay……………………………………………………………………*(page 15)*
4. **Supplementary Fig. 4** Forest plot of functional outcomes by modified Rankin Scale (mRS) (good (mRS 0–2) and favorable (mRS 0–3)) ……………..……...….*(page 16)*
5. **Supplementary Fig. 5** Sensitivity analysis by Leave-one-out plot for mean change in hematoma thickness…………………………………………………..……*(page 17)*
6. **Supplementary Fig. 6** Forest plot of subgrouping for infections & infestations and total adverse events.………………………………………………….…*(page 18)*

7. **Supplementary Fig. 7** Sensitivity analysis by Leave-one-out plot for serious adverse events……………………………………………………...….……....*(page 19)*
8. **Supplementary Fig. 8** Forest plot of subgrouping for intracranial hemorrhage and total death within 90 days……….………………………………………………*(page 20)*
9. **Supplementary Fig. 9** Trial sequential analysis for total recurrence of hematoma…………………………………………………………......….*(page 21)*
10. **Supplementary Fig. 10** Trial sequential analysis of surgical rescue….……*(page 22)*

Supplementary Table 1 PRISMA 2020 checklist.

Section and Topic	Item #	Checklist item	Location where item is reported
**TITLE**			
Title	1	Identify the report as a systematic review.	Title page
**ABSTRACT**			
Abstract	2	See the PRISMA 2020 for Abstracts checklist.	Page 2
**INTRODUCTION**			
Rationale	3	Describe the rationale for the review in the context of existing knowledge.	Page 3
Objectives	4	Provide an explicit statement of the objective(s) or question(s) the review addresses.	Page 3
**METHODS**			
Eligibility criteria	5	Specify the inclusion and exclusion criteria for the review and how studies were grouped for the syntheses.	Page 4, subsection 2.3
Information sources	6	Specify all databases, registers, websites, organisations, reference lists and other sources searched or consulted to identify studies. Specify the date when each source was last searched or consulted.	Page 4, subsection 2.2
Search strategy	7	Present the full search strategies for all databases, registers, and websites, including any filters and limits used.	Page 4, subsection 2.2 Sup material, table S2
Selection process	8	Specify the methods used to decide whether a study met the inclusion criteria of the review, including how many reviewers screened each record and each report retrieved, whether they worked independently, and if applicable, details of automation tools used in the process.	Page 5, subsection 2.4
Data collection process	9	Specify the methods used to collect data from reports, including how many reviewers collected data from each report, whether they worked independently, any processes for obtaining or confirming data from study investigators, and if applicable, details of automation tools used in the process.	Page 5, subsection 2.5
Data items	10a	List and define all outcomes for which data were sought. Specify whether all results that were compatible with each outcome domain in each study were sought (e.g. for all measures, time points, analyses), and if not, the methods used to decide which results to collect.	Page 5, subsection 2.5
	10b	List and define all other variables for which data were sought (e.g. participant and intervention characteristics, funding sources). Describe any assumptions made about any missing or unclear information.	Page 5, subsection 2.5
Study risk of bias assessment	11	Specify the methods used to assess risk of bias in the included studies, including details of the tool(s) used, how many reviewers assessed each study and whether they worked independently, and if applicable, details of automation tools used in the process.	Page 5, subsection 2.6
Effect measures	12	Specify for each outcome the effect measure(s) (e.g. risk ratio, mean difference) used in the synthesis or presentation of results.	Page 6, subsection 2.7
Synthesis methods	13a	Describe the processes used to decide which studies were eligible for each synthesis (e.g. tabulating the study intervention characteristics and comparing against the planned groups for each synthesis (item #5)).	Page 6, subsection 2.7
	13b	Describe any methods required to prepare the data for presentation or synthesis, such as handling of missing summary statistics, or data conversions.	Page 6, subsection 2.7
	13c	Describe any methods used to tabulate or visually display results of individual studies and syntheses.	Page 6, subsection 2.7
	13d	Describe any methods used to synthesize results and provide a rationale for the choice(s). If meta-analysis was performed, describe the model(s), method(s) to identify the presence and extent of statistical heterogeneity, and software package(s) used.	Page 6, subsection 2.7
	13e	Describe any methods used to explore plausible causes of heterogeneity among study	Page 6,

Section and Topic	Item #	Checklist item	Location where item is reported
		results (e.g. subgroup analysis, meta-regression).	subsection 2.7
	13f	Describe any sensitivity analyses conducted to assess robustness of the synthesized results.	Page 6, subsection 2.7
Reporting bias assessment	14	Describe any methods used to assess risk of bias due to missing results in a synthesis (arising from reporting biases).	Not applicable
Certainty assessment	15	Describe any methods used to assess certainty (or confidence) in the body of evidence for an outcome.	Page 6, subsection 2.6
**RESULTS**			
Study selection	16a	Describe the results of the search and selection process, from the number of records identified in the search to the number of studies included in the review, ideally using a flow diagram.	Page 8, subsection 3.1
	16b	Cite studies that might appear to meet the inclusion criteria, but which were excluded, and explain why they were excluded.	Page 8, subsection 3.1
Study characteristics	17	Cite each included study and present its characteristics.	Table 1 & 2, Page 8, subsection 3.2
Risk of bias in studies	18	Present assessments of risk of bias for each included study.	Page 8, subsection 3.3
Results of individual studies	19	For all outcomes, present, for each study: (a) summary statistics for each group (where appropriate) and (b) an effect estimates and its precision (e.g. confidence/credible interval), ideally using structured tables or plots.	Pages 8-9, subsections 3.4-3.5
Results of syntheses	20a	For each synthesis, briefly summarise the characteristics and risk of bias among contributing studies.	Page 12, subsection 3.3
	20b	Present results of all statistical syntheses conducted. If meta-analysis was done, present for each the summary estimate and its precision (e.g. confidence/credible interval) and measures of statistical heterogeneity. If comparing groups, describe the direction of the effect.	Pages 8-9, subsections 3.4-3.5
	20c	Present results of all investigations of plausible causes of heterogeneity among study results.	Pages 8-9, subsections 3.4-3.5
	20d	Present results of all sensitivity analyses conducted to assess the robustness of the synthesized results.	Pages 8-9, subsections 3.4-3.5
Reporting biases	21	Present assessments of risk of bias due to missing results (arising from reporting biases) for each synthesis assessed.	Page 8, subsection 3.3
Certainty of evidence	22	Present assessments of certainty (or confidence) in the body of evidence for each outcome assessed.	Page 8, subsection 3.3<br>Sup material, table S5
**DISCUSSION**			
Discussion	23a	Provide a general interpretation of the results in the context of other evidence.	Page 11
	23b	Discuss any limitations of the evidence included in the review.	Page 13
	23c	Discuss any limitations of the review processes used.	Page 13
	23d	Discuss implications of the results for practice, policy, and future research.	Page 13,14
**OTHER INFORMATION**			
Registration and protocol	24a	Provide registration information for the review, including register name and registration number, or state that the review was not registered.	Page 4, subsection 2.1
	24b	Indicate where the review protocol can be accessed, or state that a protocol was not prepared.	Page 4, subsection 2.1
	24c	Describe and explain any amendments to information provided at registration or in the protocol.	Page 4, subsection 2.1

Section and Topic	Item #	Checklist item	Location where item is reported
Support	25	Describe sources of financial or non-financial support for the review, and the role of the funders or sponsors in the review.	Title page
Competing interests	26	Declare any competing interests of review authors.	Title page
Availability of data, code, and other materials	27	Report which of the following are publicly available and where they can be found template data collection forms; data extracted from included studies; data used for all analyses; analytic code; any other materials used in the review.	Title page

Supplementary Table 2 Search strategy and literature search.

Database	Restrictions	Access date	Search strategy	No of results
**PubMed**	All Feilds	23/11/2024	("subdural hematoma*" OR "chronic subdural hematoma*" OR "nonacute subdural hematoma*" OR "subacute hematoma*" OR "chronic hematoma*" OR cSDH OR NASH) AND (("middle meningeal artery" OR MMA OR endovascular OR meningeal arteries) AND ("embolization*" OR "embolisation*" OR "vascular occlusion" OR "embolize*" OR embolizes OR embolizing) OR MMAE) AND ("Clinical Trial" OR "controlled trial" OR "randomiz*" OR "randomis*" OR randomly OR RCT OR "blind*" OR "prospective*")	85
**Cochrane**	Title Abstract Keyword	23/11/2024		49
**Scopus**	Article title OR abstract	23/11/2024		127
**WOS**	All Feilds	23/11/2024		69
**Embase**	Title	23/11/2024	((subdural hematoma*) OR (chronic subdural hematoma*) OR (nonacute subdural hematoma*) OR (subacute hematoma*) OR (chronic hematoma*) OR cSDH OR NASH) AND (((middle meningeal artery) OR MMA OR endovascular OR (meningeal arteries)) AND ((embolization*) OR (embolisation*) OR (vascular occlusion) OR (embolize*) OR embolizes OR embolizing) OR MMAE) AND ((Clinical Trial) OR (controlled trial) OR (randomiz*) OR (randomis*) OR randomly OR RCT OR (blind*) OR (prospective*))	151
**Total**				481

Supplementary Table 3 Excluded studies with reasons in the full-text screening.

Study ID	Title	Reason of exclusion
**1. Sun et al., 2024 [1]**	Therapeutic efficacy of drilling drainage combined with intraoperative middle meningeal artery occlusion in the management of chronic subdural hematoma: a clinical study	Does not meet our criteria -wrong intervention
**2. Fujioka et al., 2024 [2]**	Validity of the Cost-Effectiveness of Middle Meningeal Artery Embolization Following Hematoma Evacuation for Initial Chronic Subdural Hematoma	Does not meet our criteria -wrong study design (prospective cohort)
**3. García et al., 2024 [3]**	Endovascular treatment of chronic subdural hematoma in a dual-trained neurosurgical unit: Results and proposal of a randomized controlled trial protocol	Does not meet the criteria -wrong study design (feasibility study)
**4. Duangprasert et al., 2024 [4]**	Comparison of adjunctive middle meningeal artery embolization using embosphere particles versus surgical drainage alone for the treatment of chronic subdural hematoma: A prospective study	Does not meet the criteria -wrong study design (non-randomized trial)
**5. Toro et al., 2023 [5]**	Addition of middle meningeal artery embolization to craniotomy in the treatment of symptomatic chronic subdural hematoma improves outcomes	Does not meet our criteria -abstract only
**6. Drake et al., 2022 [6]**	Swedish trial on embolization of middle meningeal artery versus surgical evacuation in chronic subdural hematoma (SWEMMA)—a national 12-month multi-center randomized controlled superiority trial with parallel group assignment, open treatment allocation and blinded clinical outcome assessment	Does not meet our criteria -incomplete data (protocol)
**7. Kellner et al, 2021 [7]**	A prospective, multi-center, randomized controlled pivotal study to evaluate the safety and effectiveness of TRUFILL® NBCA embolization of the middle meningeal artery for the treatment of subdural hematoma – The MEMBRANE study	Does not meet the criteria -abstract only
**8. Derraz et al, 2020 [8]**	Middle meningeal artery embolization as an adjuvant treatment to surgery for symptomatic chronic subdural hematoma: A pilot study assessing hematoma volume resorption	Does not meet the criteria -abstract only

Supplementary Table 4 Baseline characteristics of the included patients' hematoma lesions.

Study ID	Groups	Subdural hematoma onset, no. (%)		Side of subdural hematoma, no. (%)			Hematoma thickness at screening (mm)
		Chronic	Subacute	Left	Right	Bilateral	
Davies et al., 2024 (EMBOLISE)	Adjunctive MMAE	115 (58.4)	82 (41.6)	97 (49.2)	100 (50.8)	42 (21.3)	21.6 ±6.3
	Standard-care	116 (57.1)	87 (42.9)	111 (54.7)	92 (45.3)	37 (18.2)	21.4 ±6.2
Liu et al., 2024 (MAGIC-MT)	Adjunctive MMAE	NM	NM	211 (58.6)	149 (41.4)	69 (19.2)	22.83 ±6.33
	Standard-care	NM	NM	199 (55)	163 (45)	82 (22.7)	22.57 ±6.18
Fiorella et al.,2024 (STEM)	Adjunctive MMAE	149 (48)	0	57 (38)	66 (44)	26 (17)	18.0 ±5.8
	Standard-care	161 (52)	0	52 (32)	72 (45)	37 (23)	18.3 ±6.3
Debs et al., 2024 (Endovascular)	Adjunctive MMAE	17 (48.5)	0	11 (65)	8 (47)	NM	21 ±9.4
	Standard-care	18 (51.4)	0	14 (78)	9 (50)	NM	23 ±9.2
Lam et al., 2023	Adjunctive MMAE	16 (45.7)	0	NM	NM	9 (56.3)	21.1
	Standard-care	19 (54.3)	0	NM	NM	4 (21)	20.9
Ng et al., 2019	Adjunctive MMAE	19 (46)	0	8 (42)	8 (42)	3 (16)	14.7 ±5.4
	Standard-care	22 (54)	0	9 (41)	10 (45)	3 (14)	13.6 ±4.7

Data are presented in mean ±SD or proportions as (%). No: Number; MMAE: middle meningeal artery embolization; NM: not mentioned.

Supplementary Table 5 Remaining for grading of recommendations assessment, development, and evaluation (GRADE) evidence profile.

Certainty assessment							Study event rates (%)		Effect		Certainty
**№ of studies**	**Study design**	**RoB**	**Inconsistency**	**Indirectness**	**Imprecision**	**Others**	**MMA embolization**	**Standard care**	**Relative (95% CI)**	**Absolute (95% CI)**	
**Change in Hematoma Volume (ml)**											
3	RCTs	not serious	serious[c]	not serious	serious[b]	none	547	567	-	MD **0.16 ml lower** (0.4 lower to 0.07 higher)	⊕⊕◯◯ Low[b,c]
**Mean days of hospital stay (days)**											
2	RCTs	not serious	not serious	not serious	serious[b]	none	377	380	-	MD **0.12 days higher** (0.02 lower to 0.26 higher)	⊕⊕⊕◯ Moderate[b]
**Good functional Outcome (mRS 0-2)**											
3	RCTs	not serious	not serious	not serious	serious[d]	none	491/554 (88.6%)	499/564 (88.5%)	**RR 1.00** (0.96 to 1.04)	**0 fewer per 1,000** (from 35 fewer to 35 more)	⊕⊕⊕◯ Moderate[d]
**Favorable functional Outcome (mRS 0-3)**											
3	RCTs	not serious	not serious	not serious	serious[d]	none	528/553 (95.5%)	535/565 (94.7%)	**RR 1.01** (0.98 to 1.03)	**9 more per 1,000** (from 19 fewer to 28 more)	⊕⊕⊕◯ Moderate[d]
**Intracranial hemorrhage**											

Certainty assessment							Study event rates (%)		Effect		Certainty
№ of studies	Study design	RoB	Inconsistency	Indirectness	Imprecision	Others	MMA embolization	Standard care	Relative (95% CI)	Absolute (95% CI)	
4	RCTs	not serious	not serious	not serious	very serious[a]	none	3/717 (0.4%)	3/750 (0.4%)	**RR 1.07** (0.27 to 4.23)	**0 fewer per 1,000** (from 3 fewer to 13 more)	⊕⊕◯◯ Low[a]
**Stroke**											
3	RCTs	not serious	not serious	not serious	very serious[a]	none	7/701 (1.0%)	5/731 (0.7%)	**RR 1.45** (0.48 to 4.34)	**3 more per 1,000** (from 4 fewer to 23 more)	⊕⊕◯◯ Low[a]
**Disease-related death at 90 days**											
3	RCTs	not serious	not serious	not serious	very serious[a]	none	9/573 (1.6%)	4/584 (0.7%)	**RR 2.18** (0.72 to 6.65)	**8 more per 1,000** (from 2 fewer to 39 more)	⊕⊕◯◯ Low[a]
**Total Neurologic death at 180 days**											
2	RCTs	not serious	not serious	not serious	very serious[a]	none	12/341 (3.5%)	8/369 (2.2%)	**RR 1.59** (0.65 to 3.86)	**13 more per 1,000** (from 8 fewer to 62 more)	⊕⊕◯◯ Low[a]

CI: confidence interval; **MD:** mean difference; **RR:** risk ratio; **RoB:** risk of bias; **MMA:** middle meningeal artery; **RCT**: randomized controlled trial.

Explanations

a. Wide CI with crossing no effect line (1.0), not exclude the risk of appreciable benefit/harm.

b. Cross the line of no effect (0), not exclude the risk of appreciable benefit/harm.

c. $I^2 \geq 50\%$; shows significant heterogeneity.

d. Cross no effect line (1.0), not exclude the risk of appreciable benefit/harm.

e. Wide CI, which may not exclude the risk of appreciable benefit/harm.

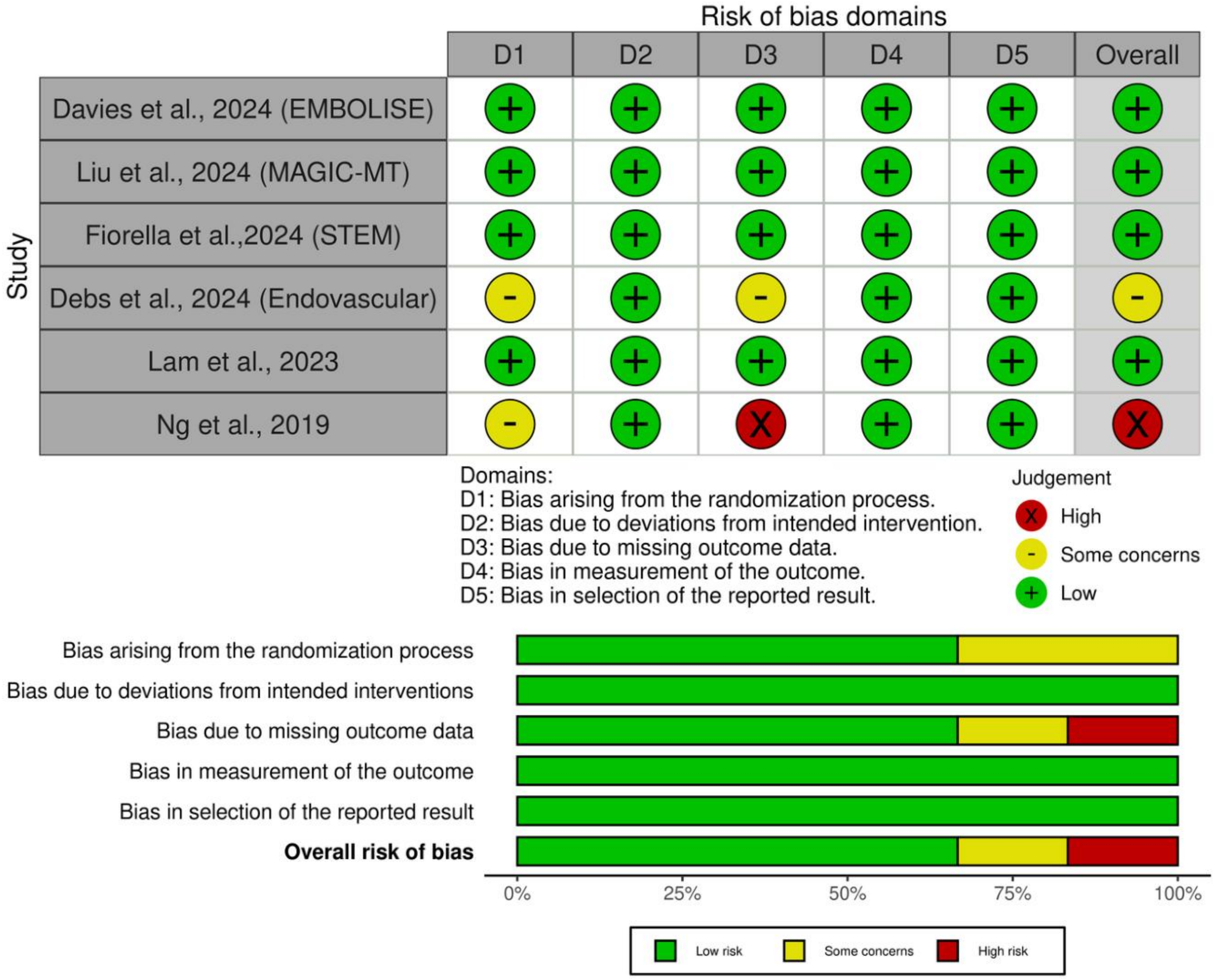

Supplementary Fig. 1 Overview of the risk of bias of the included randomized controlled trials.

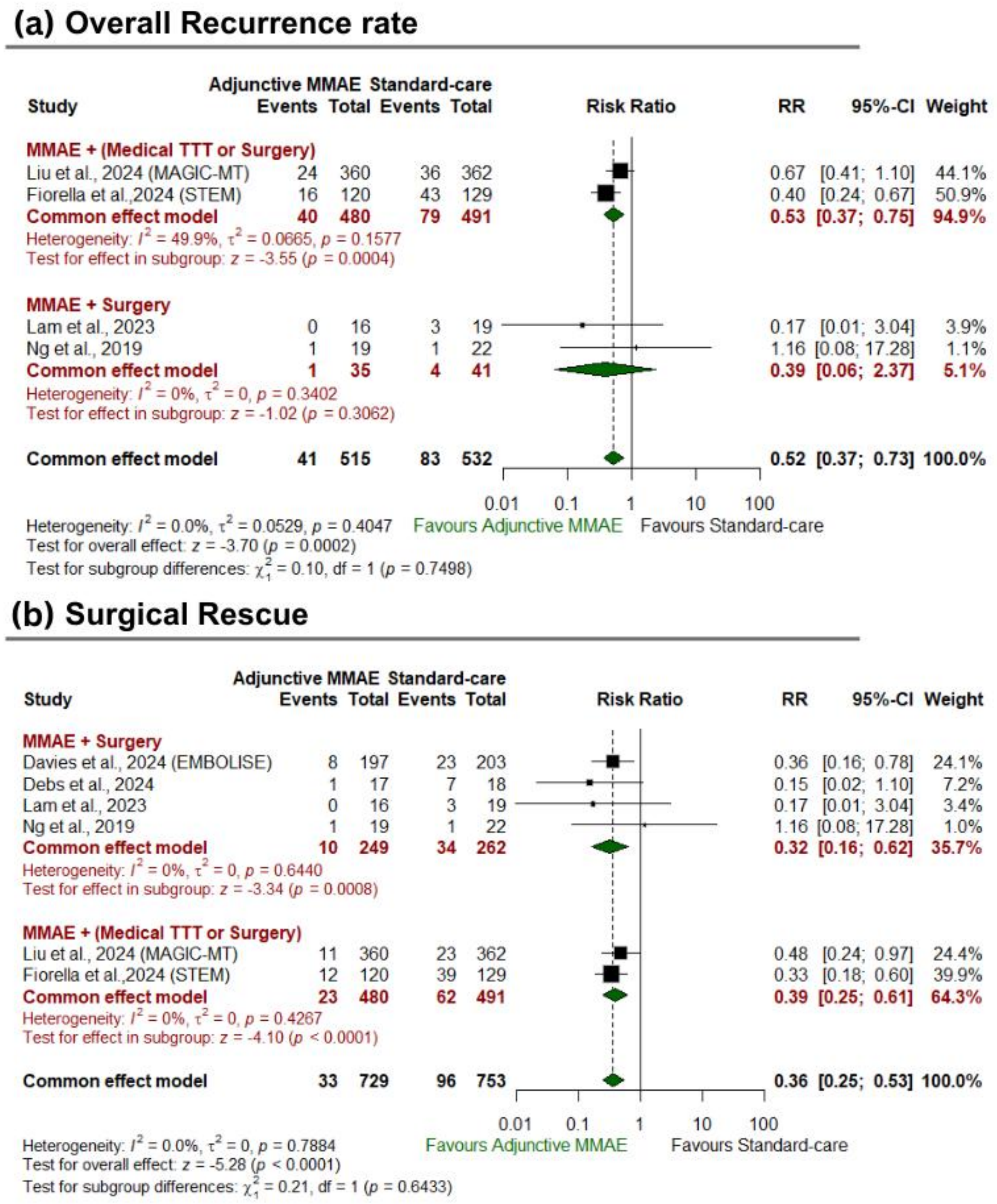

Supplementary Fig. 2 Forest plot of subgrouping for overall recurrence rate and surgical rescue. *MMAE* middle meningeal artery embolization *RR* risk ratio *CI* Confidence interval.

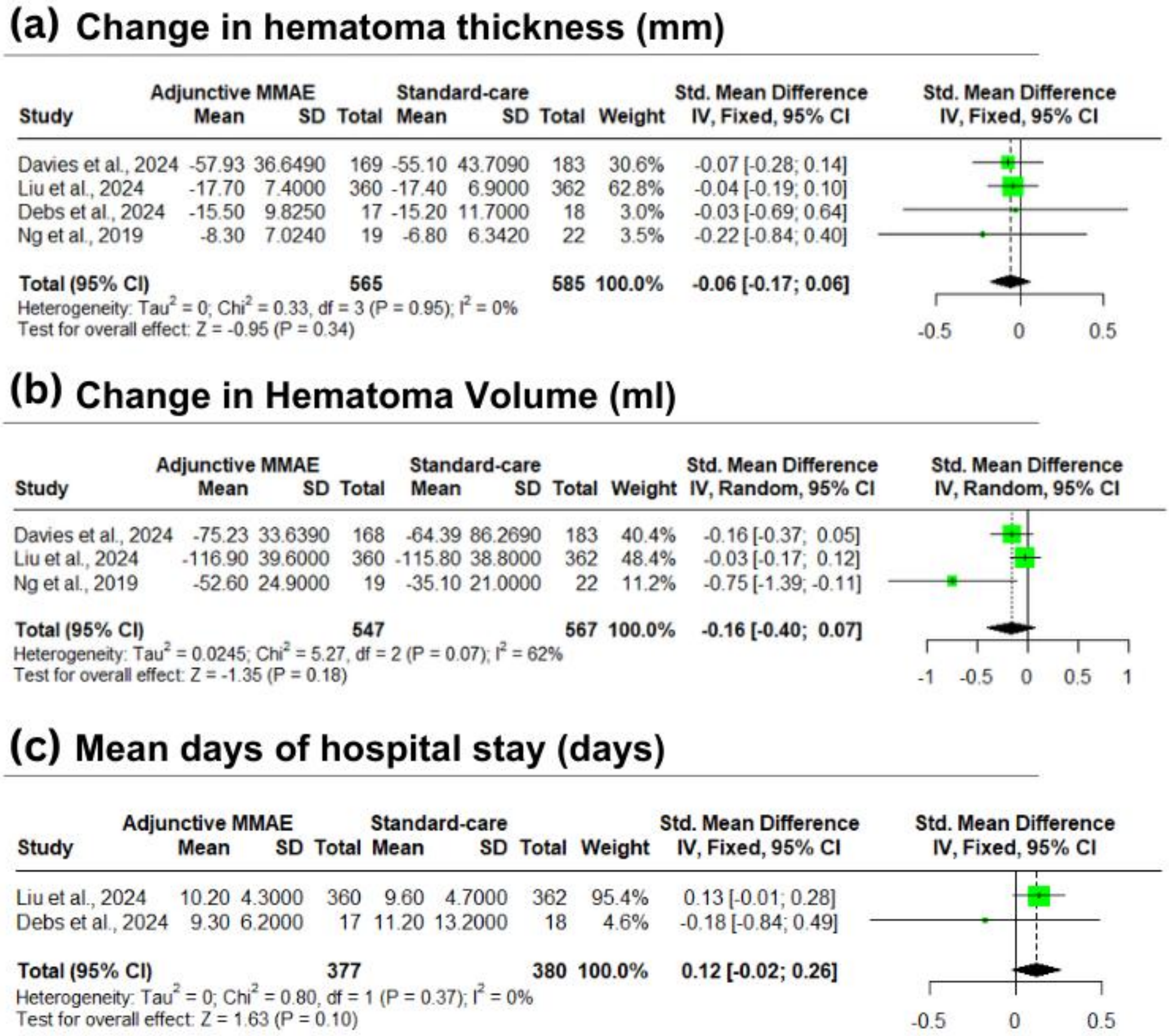

Supplementary Fig. 3 Forest plot of radiological efficacy outcomes and hospital stay. MMAE middle meningeal artery embolization, Std standardized, CI Confidence interval.

(a) Good functional Outcome (mRS 0-2)

Study	Adjunctive MMAE Events	Adjunctive MMAE Total	Standard-care Events	Standard-care Total	Weight	Risk Ratio MH, Fixed, 95% CI
Davies et al., 2024	144	177	154	184	30.5%	0.97 [0.88; 1.07]
Liu et al., 2024	335	360	333	362	67.1%	1.01 [0.97; 1.05]
Debs et al., 2024	12	17	12	18	2.4%	1.06 [0.68; 1.66]
**Total (95% CI)**	**491**	**554**	**499**	**564**	**100.0%**	**1.00 [0.96; 1.04]**

Heterogeneity: $Tau^2 = 0$; $Chi^2 = 0.62$, df = 2 (P = 0.73); $I^2 = 0\%$
Test for overall effect: Z = 0.03 (P = 0.98)

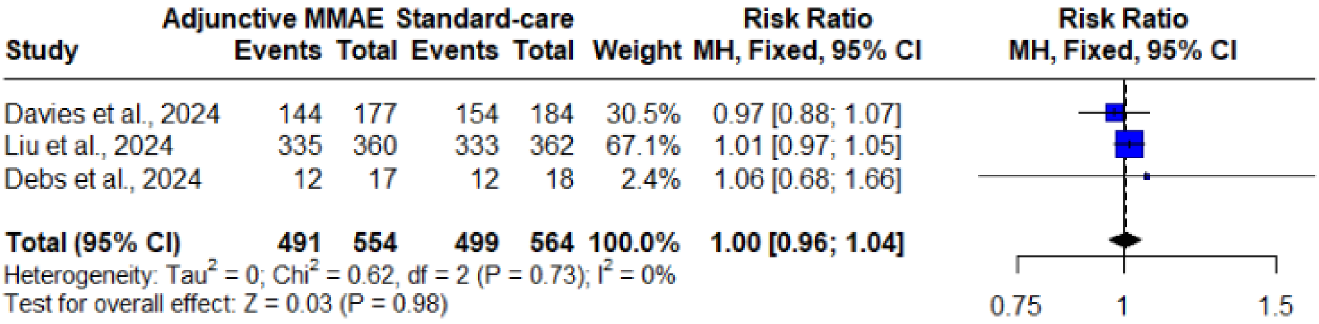

(b) Favorable functional Outcome (mRS 0-3)

Study	Adjunctive MMAE Events	Adjunctive MMAE Total	Standard-care Events	Standard-care Total	Weight	Risk Ratio MH, Fixed, 95% CI
Davies et al., 2024	160	177	170	184	31.5%	0.98 [0.92; 1.04]
Liu et al., 2024	352	360	349	362	65.7%	1.01 [0.99; 1.04]
Lam et al., 2023	16	16	16	19	2.9%	1.18 [0.98; 1.43]
**Total (95% CI)**	**528**	**553**	**535**	**565**	**100.0%**	**1.01 [0.98; 1.03]**

Heterogeneity: $Tau^2 < 0.0001$; $Chi^2 = 3.70$, df = 2 (P = 0.16); $I^2 = 46\%$
Test for overall effect: Z = 0.56 (P = 0.58)

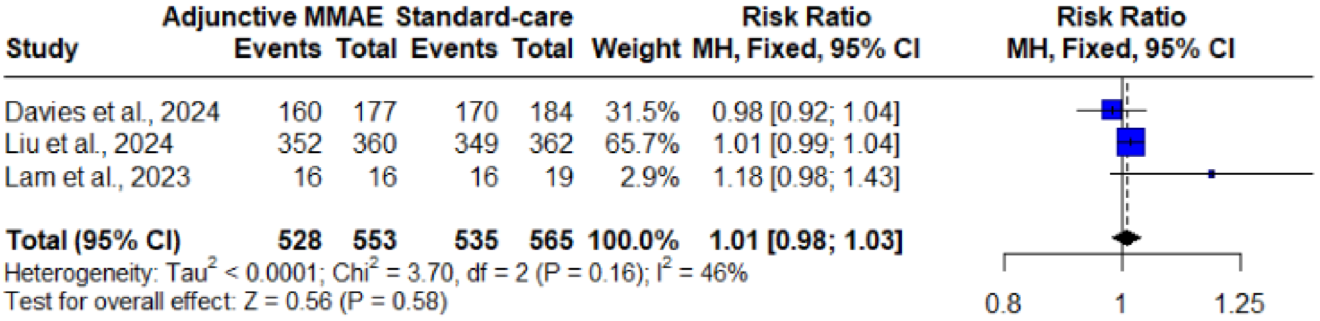

Supplementary Fig. 4 Forest plot of functional outcomes by modified Rankin Scale (mRS) (good (mRS 0–2) and favorable (mRS 0–3)). CI Confidence interval.

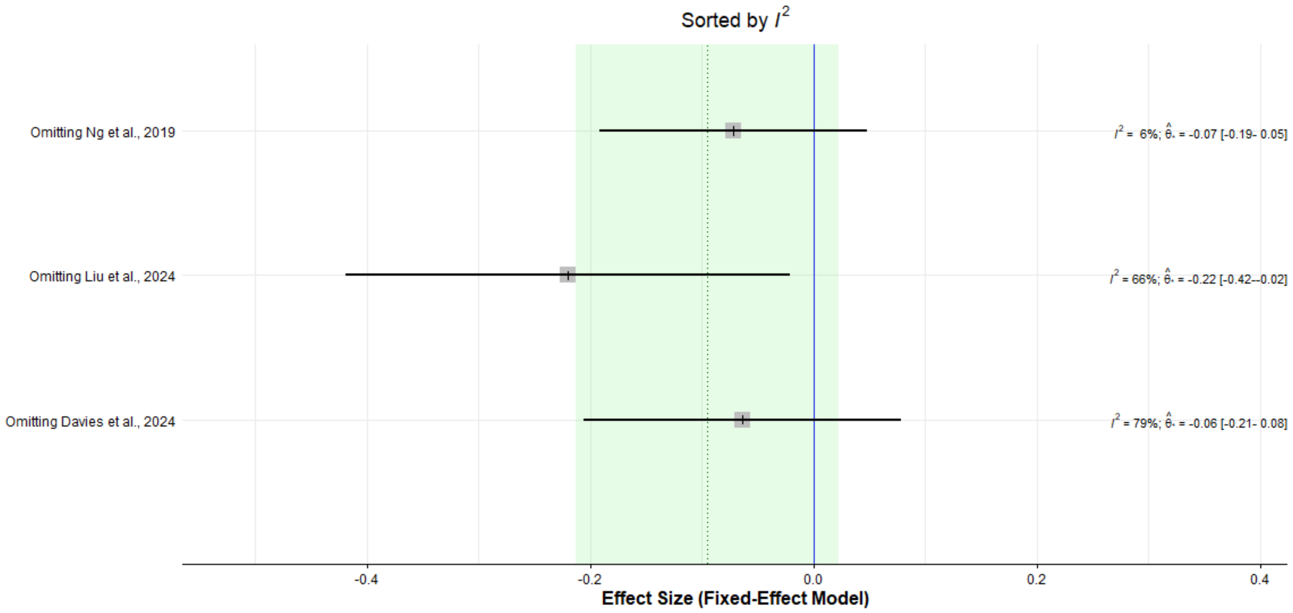

Supplementary Fig. 5 Sensitivity analysis by leave-one-out plot for mean change in hematoma thickness.

(a) Infections and Infestations

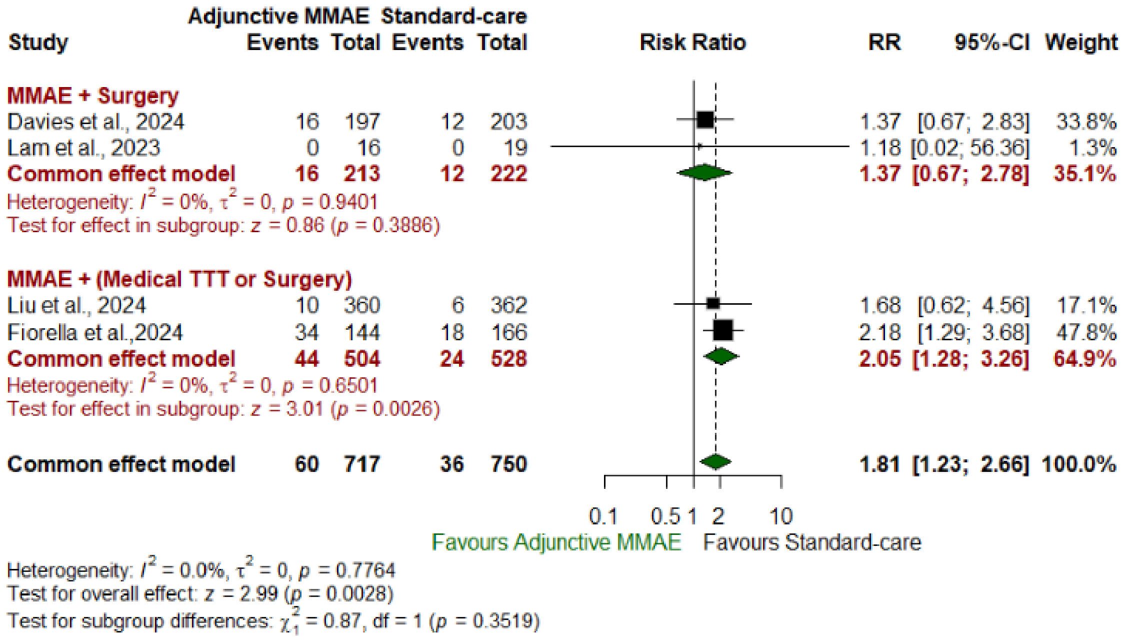

(b) Total Adverse Events

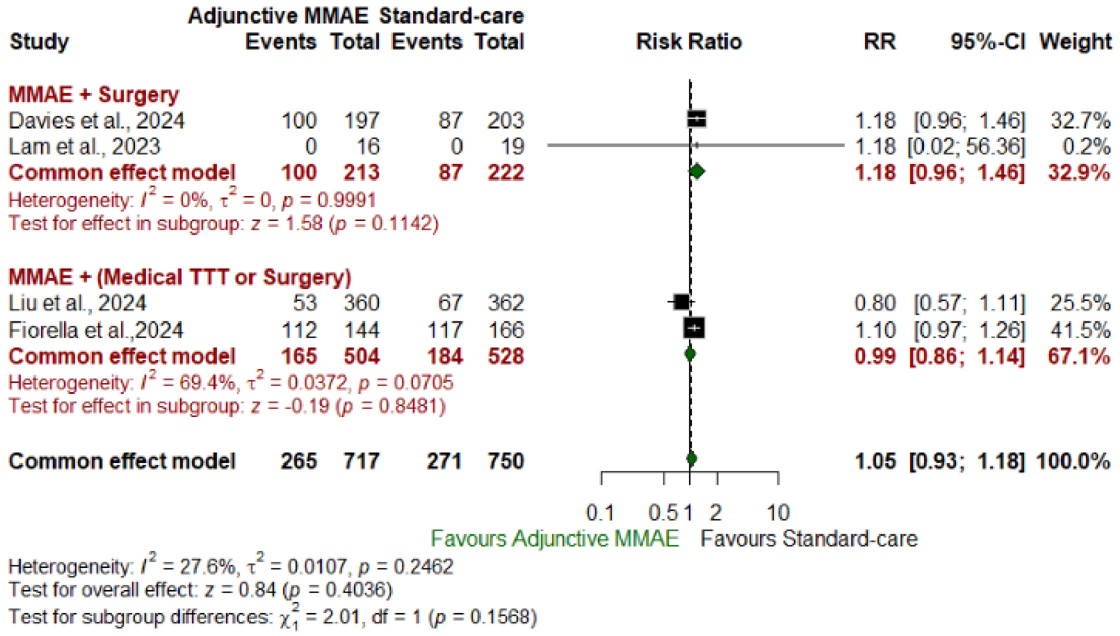

Supplementary Fig. 6 Forest plot of subgrouping for intections & infestations and total adverse events. *MMAE* middle meningeal artery embolization *RR* risk ratio *CI* Confidence interval.

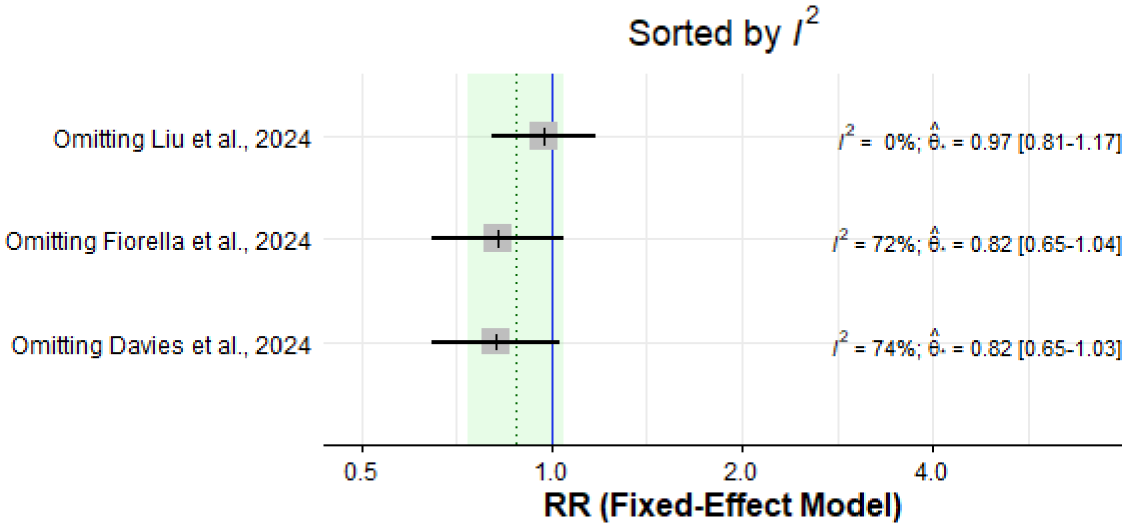

Supplementary Fig. 7 Sensitivity analysis by leave-one-out plot for serious adverse events. *RR* risk ratio.

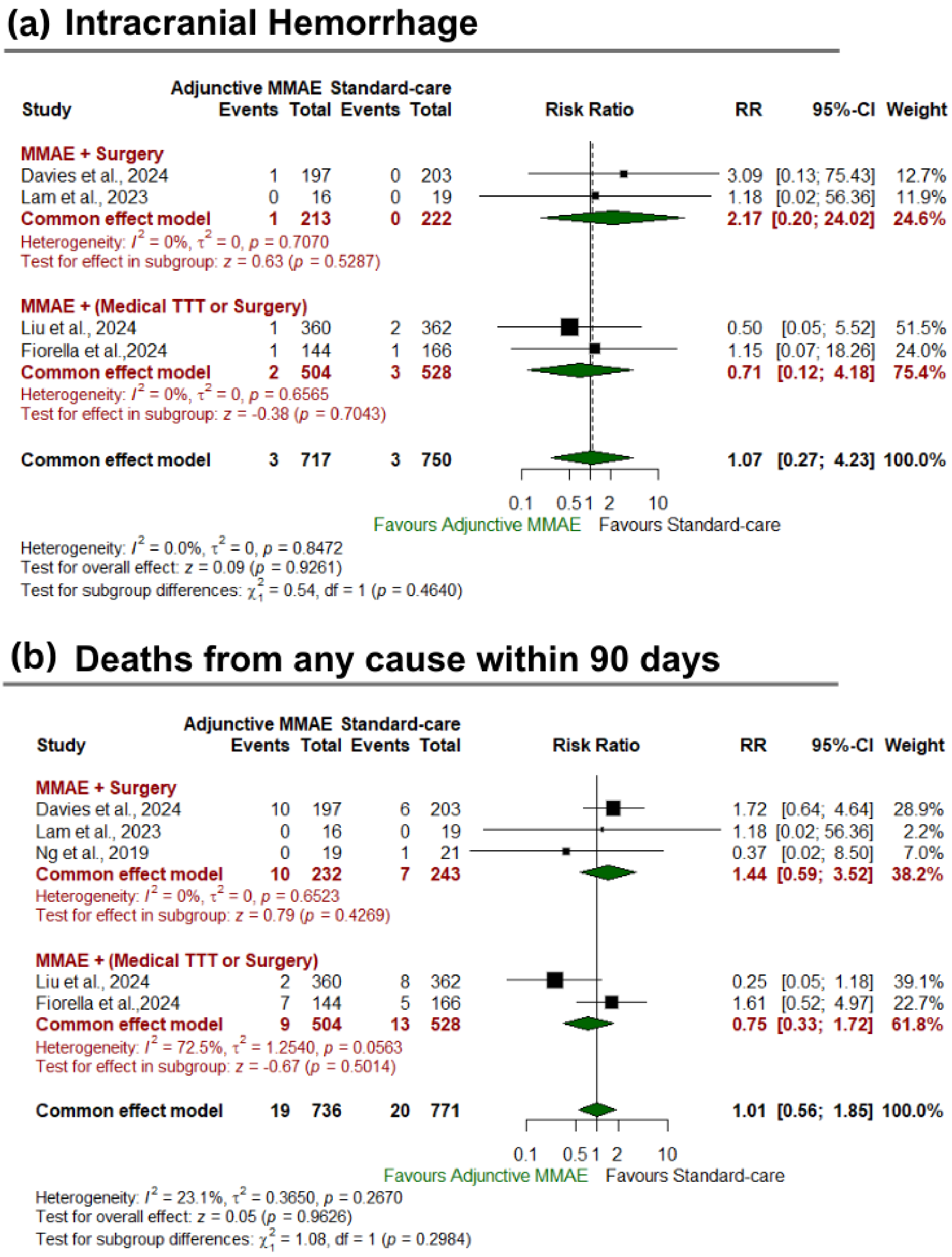

Supplementary Fig. 8 Forest plot of subgrouping for intracranial hemorrhage and total death within 90 days. *MMAE* middle meningeal artery embolization *RR* risk ratio *CI* Confidence interval.

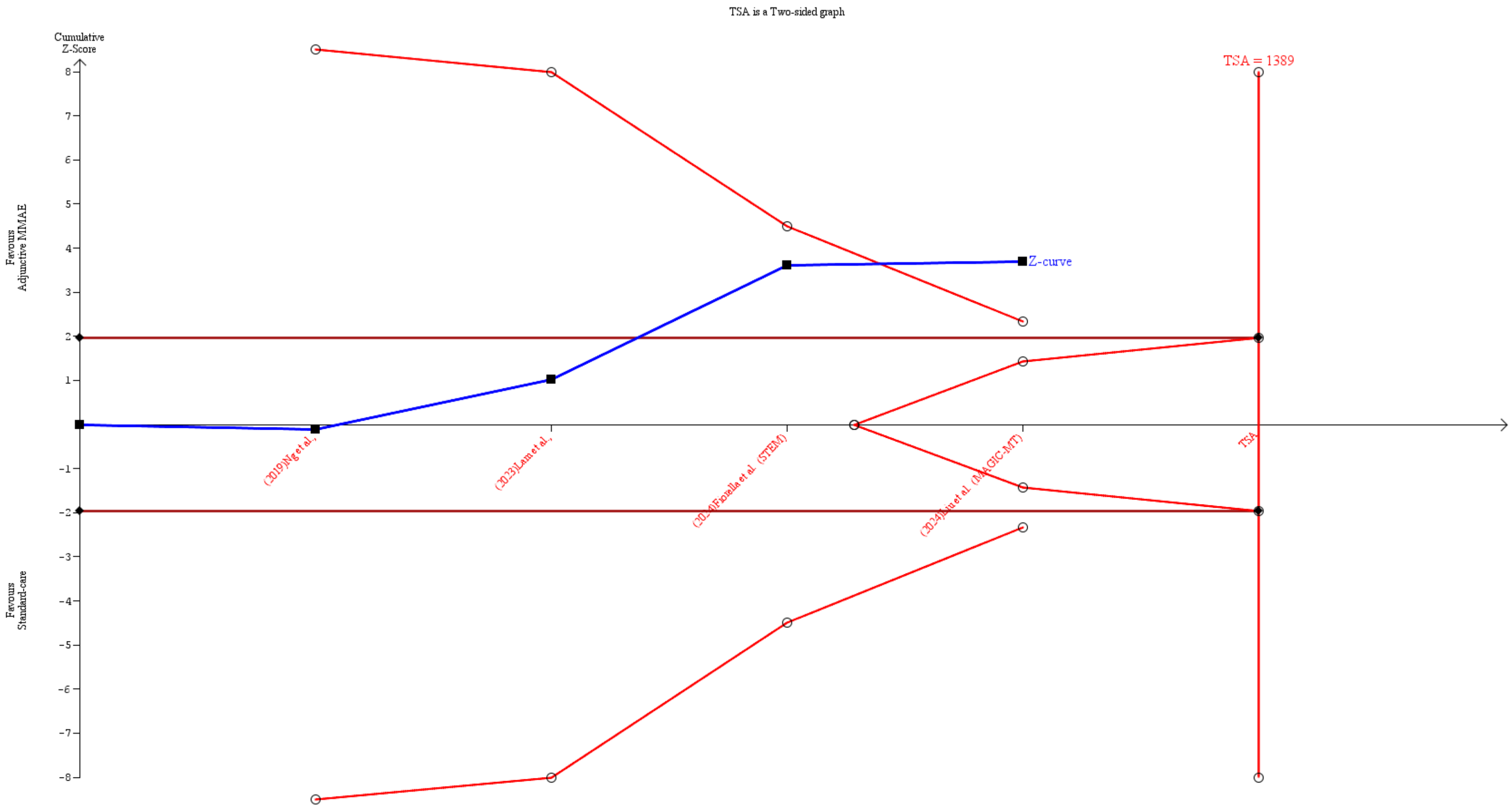

Supplementary Fig. 9 Trial sequential analysis for total recurrence of hematoma.

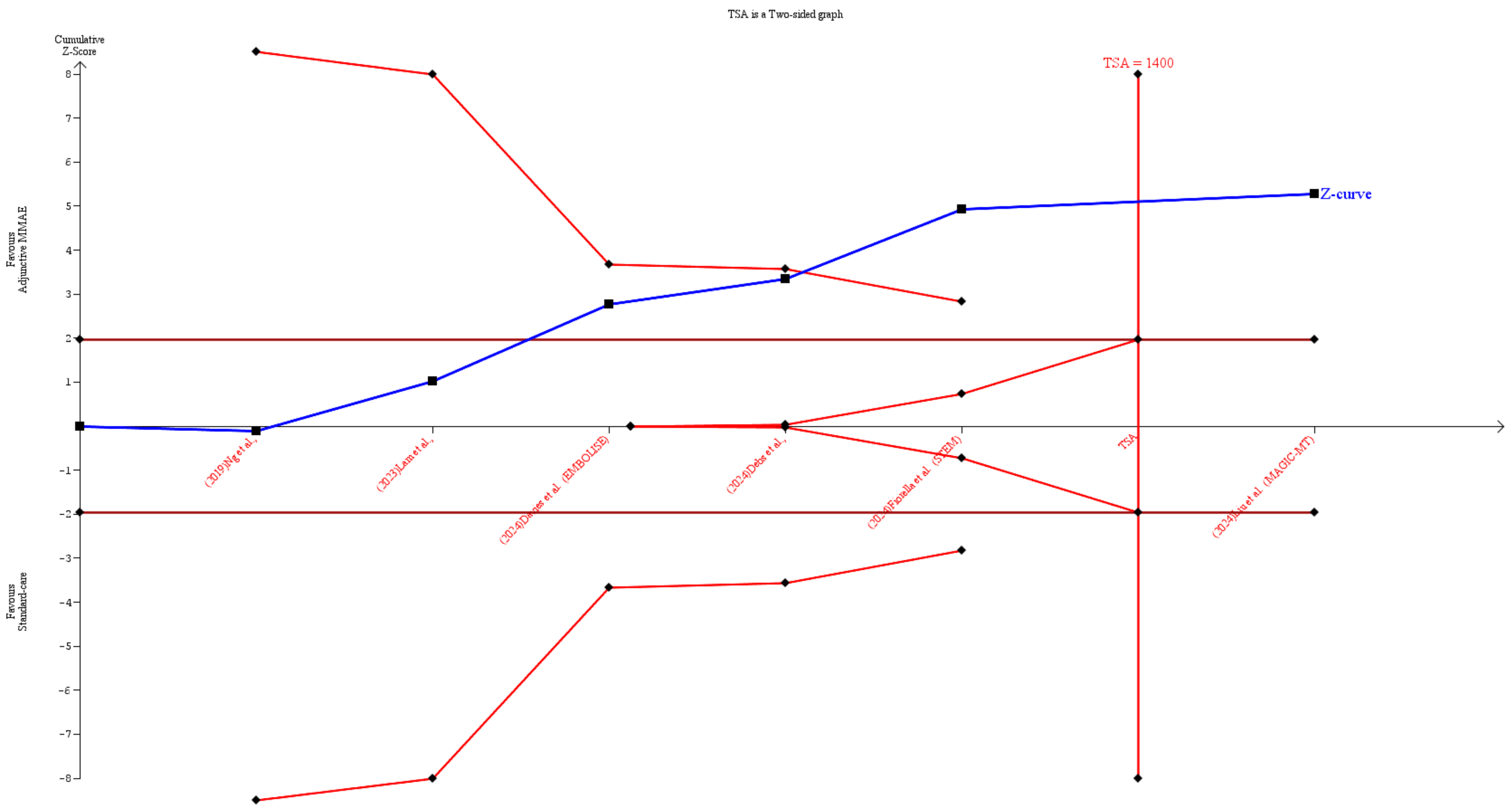

Supplementary Fig. 10 Trial sequential analysis for surgical rescue.

References.

[1]. Sun T, Shao D, Li J, et al. Therapeutic efficacy of drilling drainage combined with intraoperative middle meningeal artery occlusion in the management of chronic subdural hematoma: a clinical study. *Neurosurg Rev.* 2024;47:293.

[2]. Fujioka M, Ishii Y, Chiba K, et al. Validity of the cost-effectiveness of middle meningeal artery embolization following hematoma evacuation for initial chronic subdural hematoma. *World Neurosurg.* 2024;190:e175-e180.

[3]. García SG, Regañón IA, Chafla SC, Herrero RS. Endovascular treatment of chronic subdural hematoma in a dual-trained neurosurgical unit: results and proposal of a randomized controlled trial protocol. *Neurocir (English Ed).* Published Online First: 2024.

[4]. Duangprasert G, Sukhor S, Noiphithak R, Tantongtip D. Comparison of adjunctive middle meningeal artery embolization using embosphere particles versus surgical drainage alone for the treatment of chronic subdural hematoma: a prospective study. *J Clin Neurosci.* 2024;128:110808.

[5]. Toro D, Debs LV. Addition of middle meningeal artery embolization to craniotomy in the treatment of symptomatic chronic subdural hematoma improves outcomes. *J Am Coll Surg.* 2023;237(5 Suppl). doi:10.1097/XCS.0000000000000833.

[6]. Drake M, Ullberg T, Nittby H, Marklund N, Wassélius J. Swedish trial on embolization of middle meningeal artery versus surgical evacuation in chronic subdural hematoma (SWEMMA)—a national 12-month multi-center randomized controlled superiority trial with parallel group assignment, open treatment allocation. *Trials.* 2022;23:926. doi:10.1186/s13063-022-06842-4.

[7]. Kellner C, Al-Mufti F, Gupta R, Jankowitz B, Starke R, Rai A. A prospective, multi-center, randomized controlled pivotal study to evaluate the safety and effectiveness of trufill® NBCA embolization of the middle meningeal artery for the treatment of subdural hematoma–The membrane study. Published Online First: 2021.

[8]. Derraz I, Ng S, Boetto J, et al. Middle meningeal artery embolization as an adjuvant treatment to surgery for symptomatic chronic subdural hematoma: a pilot study assessing hematoma volume resorption. *J Neuroradiol.* 2020;47:78. doi:10.1016/j.neurad.2019.12.027.
